# Visualization of Shared Genomic Regions and Meiotic Recombination in High-Density SNP Data

**DOI:** 10.1371/journal.pone.0006711

**Published:** 2009-08-21

**Authors:** Elisha D. O. Roberson, Jonathan Pevsner

**Affiliations:** 1 Program in Human Genetics, Johns Hopkins School of Medicine, Baltimore, Maryland, United States of America; 2 Department of Neurology, Hugo W. Moser Research Institute at Kennedy Krieger, Baltimore, Maryland, United States of America; 3 Department of Neuroscience, Johns Hopkins School of Medicine, Baltimore, Maryland, United States of America; Texas A&M University, United States of America

## Abstract

**Background:**

A fundamental goal of single nucleotide polymorphism (SNP) genotyping is to determine the sharing of alleles between individuals across genomic loci. Such analyses have diverse applications in defining the relatedness of individuals (including unexpected relationships in nominally unrelated individuals, or consanguinity within pedigrees), analyzing meiotic crossovers, and identifying a broad range of chromosomal anomalies such as hemizygous deletions and uniparental disomy, and analyzing population structure.

**Principal Findings:**

We present SNPduo, a command-line and web accessible tool for analyzing and visualizing the relatedness of any two individuals using identity by state. Using identity by state does not require prior knowledge of allele frequencies or pedigree information, and is more computationally tractable and is less affected by population stratification than calculating identity by descent probabilities. The web implementation visualizes shared genomic regions, and generates UCSC viewable tracks. The command-line version requires pedigree information for compatibility with existing software and determining specified relationships even though pedigrees are not required for IBS calculation, generates no visual output, is written in portable C++, and is well-suited to analyzing large datasets. We demonstrate how the SNPduo web tool identifies meiotic crossover positions in siblings, and confirm our findings by visualizing meiotic recombination in synthetic three-generation pedigrees. We applied SNPduo to 210 nominally unrelated Phase I / II HapMap samples and, consistent with previous findings, identified six undeclared pairs of related individuals. We further analyzed identity by state in 2,883 individuals from multiplex families with autism and identified a series of anomalies including related parents, an individual with mosaic loss of chromosome 18, an individual with maternal heterodisomy of chromosome 16, and unexplained replicate samples.

**Conclusions:**

SNPduo provides the ability to explore and visualize SNP data to characterize the relatedness between individuals. It is compatible with, but distinct from, other established analysis software such as PLINK, and performs favorably in benchmarking studies for the analyses of genetic relatedness.

## Introduction

High-density single nucleotide polymorphism (SNP) genotyping is used in association studies to find markers linked to loci that contribute to human disease and variation. Data derived from this approach have led to the identification of candidate loci in several human diseases, including macular degeneration [1], [2], rheumatoid arthritis [3], [4], and breast cancer [5]–[8]. SNP data are also useful for the analysis of homozygosity and copy number alterations in individuals, and inheritance patterns in pedigrees [9]. Two principles central to SNP use in association studies are identity by state (IBS) and identity by descent (IBD). IBS is the sharing of alleles between individuals. IBD is the sharing of alleles between individuals with an identified, common ancestral source of the alleles. Consider a family with a father and mother having genotypes CC and AC, respectively, and two children with an AC genotype. Each sibling's A allele is shared IBD since it must have been inherited from the mother on the same physical chromosome. The C alleles, however, are shared IBS between the siblings. One of the father's two C alleles was transmitted to each sibling, but it is not possible to discern whether the C alleles were derived from the same physical chromosome.

There are many applications of IBD studies including the affected sib-pair method [10], tests for linkage [11], [12] and studies of genetic relatedness [13]. The Merlin software package is commonly used for linkage studies, and includes IBD calculations [14]. The use of IBD requires knowledge of the relationship between individuals, or population allele frequencies to calculate IBD probabilities. In contrast, IBS can be calculated without knowledge of pedigree structure and does not require allele frequency information. In this paper we describe the SNPduo software tools. SNPduo is available in command-line and web accessible versions, referred to as SNPduo++ and SNPduo, respectively. SNPduo provides a method for the visualization of IBS between two individuals in an intuitive and informative way. By analyzing and plotting the IBS states between two individuals by the chromosomal location of each SNP, blocks of shared (and unshared) chromosomal material can be located. SNPduo++ provides a method for analyzing the mean and standard deviation of IBS states in large datasets, facilitating the discovery of unexpected relationships and population structure. Furthermore we show how IBS analysis in families can visualize meiotic crossover points in siblings (supported with synthetic data), delineate regions of hemizygous deletion, and detect uniparental disomy based upon discrepant IBS patterns. SNPduo identifies this broad range of genetic phenomena because it relies on high-density SNP data which are now routinely available. As the size of data sets grow in terms of the number of samples, so does the possibility of misclassified relationships due non-paternity, sample mislabeling, unexpected relatedness within families, and the unintentional inclusion of multiple family members (possibly recruited at different times or at different centers). SNPduo can help identify such errors.

## Results

### SNPduo demonstrates interpretable patterns of allele sharing

We developed the SNPduo program that tabulates and visualizes genetic relatedness based on SNP genotypes between pairs of individuals. The tool is web-accessible at http:// pevsnerlab.kennedykrieger.org/SNPduo. In particular, at each SNP we consider the sharing of 0, 1, or 2 alleles IBS. SNPduo requires genotype input, but is not a genotyping algorithm. Therefore the accuracy of the results depends upon the accuracy of the genotyping algorithm i.e. how often the observed genotype is the true genotype. For this reason all of the IBS classifications and genotypes described in this publication were the observed states. We determine the chromosomal locations at which a given SNP shared both alleles between two individuals (AA to AA, AB to AB, or BB to BB; IBS-2), one allele (e.g. AA to AB or AB to BB; IBS-1), or no alleles (AA to BB or BB to AA; IBS-0).

**Figure 1:**
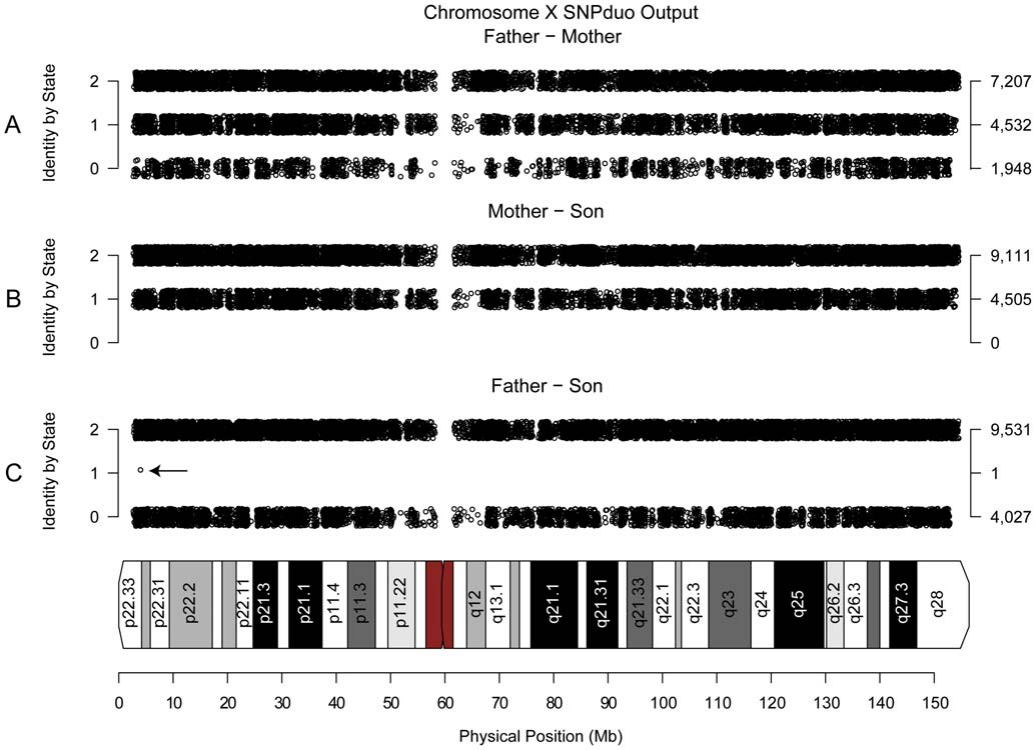
IBS patterns for father, mother, and son on chromosome X. A portion of the SNPduo output for three pairwise comparisons of the X chromosome of father/mother (A), mother/son (B), and father/son (C) genotyped on the Illumina HumanHap 550K platform. In the unrelated parents, there were many instances of no shared alleles (e.g. AA to BB; panel A). In the mother-son comparison, there were no IBS-0 SNPs because the son inherited a copy of the maternal X. In the father/son comparison, each chromosome was hemizygous (either A or B genotypes, interpreted as AA or BB) and in the absence of heterozygous calls no IBS-1 SNPs were expected to occur since the X chromosomes were non-identical (both IBS-2 and IBS-0 SNPs were apparent). Thus, the one call of an IBS-1 SNP (arrow) was likely a genotyping error. The standard SNPduo output includes genotype calls (not shown; see e.g. Fig. 3).

The input to SNPduo is a set of genotype calls with annotated chromosomes and physical map positions; the output includes an image with series of tracks plotting single-point, pairwise IBS by physical map position, a text summary file, and an IBS block file viewable on the UCSC genome browser. Images for three pairwise comparisons are shown for the X chromosome of a trio (father, mother, and son, Fig. 1). The unrelated parents included many IBS-0 calls, typical of genotypes from unrelated individuals (Fig. 1a). The mother/son pairwise comparison included no IBS-0 calls since she transmitted one of her two X chromosomes to the son (Fig. 1b). Notably, although the male X was hemizygous (and had genotypes A or B), current SNP genotyping algorithms typically generate all biallelic calls (AA or BB). Therefore even though the son's genotype is interpreted as diploid he always shared at least one observed allele with the mother's genotype. The father/son comparison showed almost no IBS-1 calls (Fig. 1c). These two chromosomes are both hemizygous and unrelated, and so include IBS-2 (e.g. AA matching AA) and IBS-0 (e.g. AA matching BB) SNPs. Rare instances of IBS-1 likely represent genotyping errors in which A or B genotypes were mistakenly identified as heterozygous AB calls. Vertical blanks with no data points on any track of a plot, including the No Call track of the individual genotypes, represent areas with no SNPs on the genotyping platform, typically in regions comprised of repetitive DNA (e.g. pericentromeric regions or areas of segmental duplications). Furthermore, it should be noted that although many areas appear to be a solid or nearly solid bar in this figure, the image is actually composed of individual SNPs plotted as dots by chromosome position with vertical jittering to make the track density easier to see.

**Figure 2:**
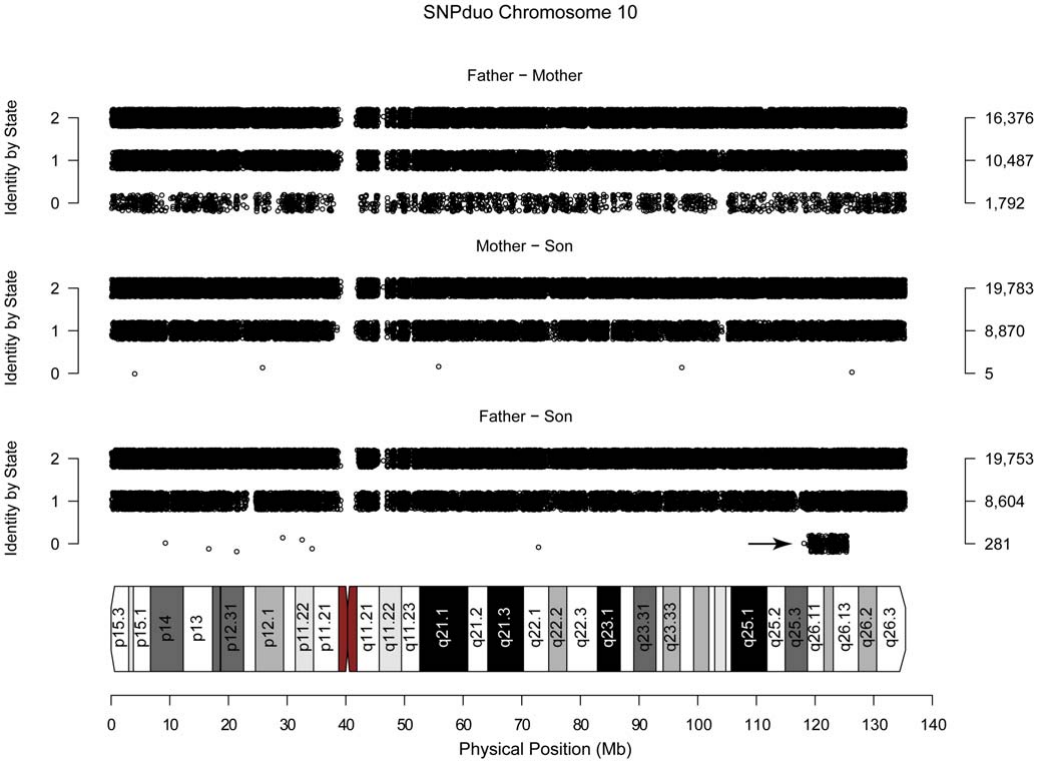
IBS patterns for parents and a child with a hemizygous deletion. A portion of the SNPduo output for chromosome 10 of father/mother (top panel), mother/son (middle panel), and father/son (bottom panel) comparisons genotyped on the Illumina HumanHap 550K platform. As expected the unrelated father and mother showed pattern of overlaid IBS-2, IBS-1, and IBS-0 tracks. Also as expected the mother and son comparison demonstrated a parent to child relationship of IBS-2 and IBS-1 tracks overlaid. The father to son comparison, however, demonstrated an additional pattern of IBS-2, IBS-1, and IBS-0 tracks overlaid (arrow). This block represents an interstitial hemizygous deletion of the paternal allele in the child.

When examining inheritance patterns in families occasionally aberrant patterns are observed. In particular, consider the pairwise comparisons of parents to a child who has a *de novo* deletion. The parents can have AA, AB, or BB genotypes for each SNP. In the region of deletion the child may have the genotype of A or B. However, genotyping algorithms interpret this as either AA or BB. For the parent whose allele was not deleted, the pattern appeared as a normal parent/child relationship with IBS-2 and IBS-1 tracks overlaid (Fig. 2, middle panel). In contrast, the parent whose allele was deleted in the child showed a pattern of IBS-2 and IBS-1 overlaid in diploid regions, but showed IBS-2, IBS-1, and IBS-0 in the region of the hemizygous deletion (Fig. 2, bottom panel arrow). In this case, the proband had an interstitial hemizygous deletion of approximately 7 megabases (Mb) as determined by karyotyping and SNP analyses [15]. The 7 Mb IBS-0 region appeared because of the genotype interpretation artifacts.

### SNPduo visualizes variably shared genomic regions in siblings

**Figure 3:**
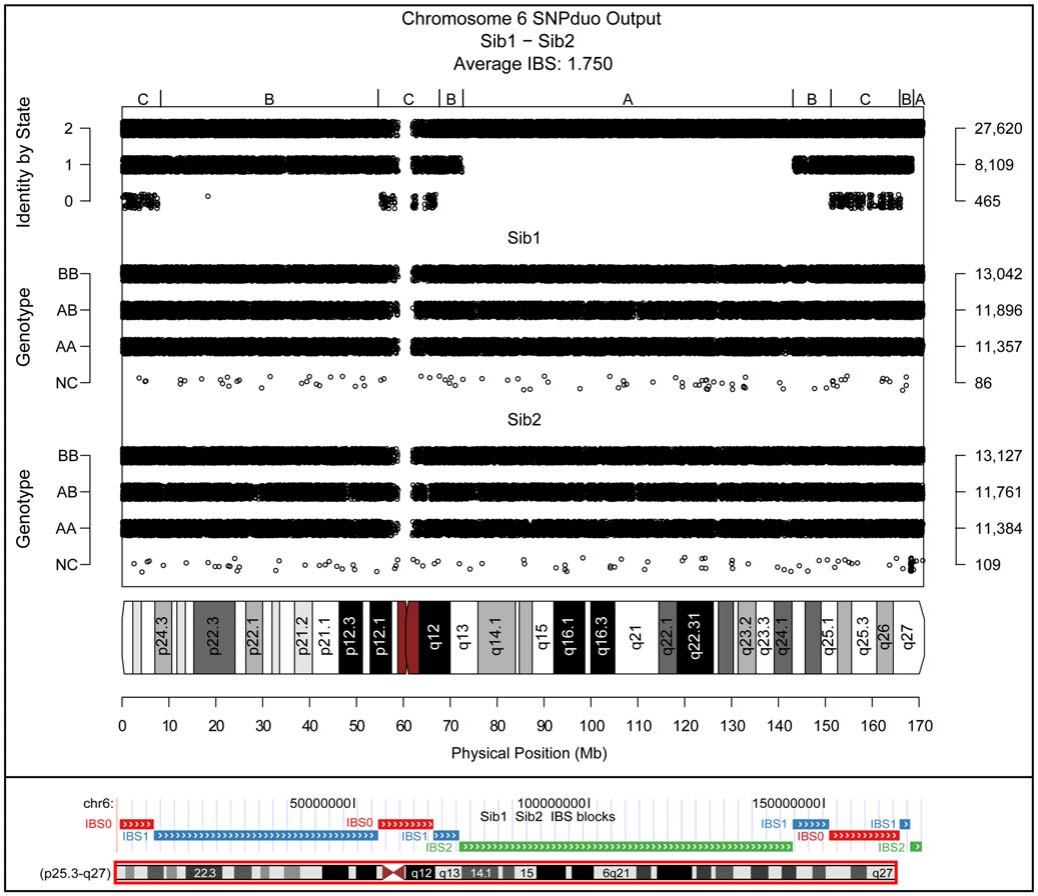
Meiotic recombination in siblings. Upper panel: SNPduo output for chromosome 6 comparing two siblings genotyped on the Illumina HumanHap 550K platform. The standard output format from the SNPduo web tool is shown. Three different patterns were apparent (see top panel). The region A pattern had IBS-2 SNPs alone. The region B pattern consisted of IBS-2 and IBS-1 tracks overlapping. Region C consisted of IBS-2, IBS-1, and IBS-0 tracks overlapping. IBS track types switched several times across the length of the chromosome, representing meiotic crossover sites. SNPduo output included plots of genotype calls for each of the two individuals compared on the middle and bottom panels (including summaries of the number of BB, AB, AA, and no call genotypes), the IBS state of each SNP on the top panel (including the number if IBS-2, IBS-1, and IBS-0 SNPs), and a chromosome ideogram at the bottom. Lower panel: Screen capture of SNPduo density segmentation viewed on the UCSC Genome Browser. The SNPduo output included a .bed file that was uploaded to the genome browser as a custom track. Block types corresponded to the SNPduo IBS output, as did the block type change points. Note that there are no SNP data across the centromere (see upper panel), but the segmentation crosses the centromere (note the IBS-0 block, lower panel) reflecting the absence of detectable crossovers in that region.

Siblings inherit genetic material from common parents, and therefore share some genetic material with each other. However, the amount of sharing between any pair of siblings for any given chromosome varies between 0% and 100%. We performed pairwise analyses of siblings and examined all chromosomes. An example of a SNPduo output comparing siblings from a previously described family [16] (Fig. 3, upper panel) showed alternating patterns of IBS-2, -1 and -0 tracks. Three IBS patterns were evident. (1) If both siblings inherit the same alleles from both parents then they share two alleles IBS, and only the IBS-2 track will be present on a SNPduo plot (Fig. 3, upper panel, regions A). (2) Now consider that sibling 1 inherits a portion of the maternal grandfather's chromosome and a portion of the paternal grandmother's chromosome, while sibling 2 inherits a portion of the maternal grandmother's chromosome (unshared between siblings) and a portion of the paternal grandmother's chromosome (shared between siblings). In this case the siblings share only one chromosome segment (paternal grandmother's chromosome), and an overlapping pattern of IBS-2 and IBS-1 is apparent (Fig. 3, upper panel, regions B). (3) Finally, sibling 1 might inherit a segment of maternal grandfather's chromosome and a segment of paternal grandmother's chromosome, while sibling 2 inherits a segment of maternal grandmother's chromosome (unshared between siblings) and a segment of paternal grandfather's chromosome (unshared between siblings). In this instance, since the siblings do not have any common ancestry in this chromosomal segment, overlapping patterns of IBS-2, IBS-1, and IBS-0 are apparent on the SNPduo plot (Fig. 3, upper panel, regions C). Note that in region C where there are zero shared alleles IBS-1 and IBS-2 patterns also occur, and in region B where there is one shared allele there are also IBS-2 SNPs. The nature of these patterns is explainable by the heterozygosity rate. To be considered a SNP (a form of polymorphism) the minor allele frequency need only be 1%. SNPs with a low minor allele frequency, especially within a population group, will always have some SNPs with identically shared (IBS-2) or partially shared (IBS-1) genotypes between individuals.

### SNPduo reveals meiotic crossover points in sibling comparisons

The sharing of chromosomal segments between siblings is characterized by alternating regions of IBS-2, IBS-1, and IBS-0. Each sibling may inherit either recombinant or non-recombinant alleles from either grandparent for each chromosome from each parent; furthermore, every gamete has a different, specific set of recombinations.

The switch between IBS track types in sibling comparisons was not random and occurred by the following pattern: IBS-2 ↔ IBS-1 ↔ IBS-0 (Fig. 3 A↔B↔C). IBS-0 → IBS-2 transitions were not seen (although the region of IBS-1 was occasionally very small). We hypothesized that the change points represented meiotic crossover events. For an IBS-2 → IBS-0 or vice versa transition without the intermediate transition to an IBS-1 track type to occur, two crossovers would have to occur in a region between SNPs. It is worth clarifying that these transition patterns apply only to the overall IBS track type in a region, not the individual SNP interpretations. Consecutive single point SNP calls transition freely between IBS classes. The SNPduo web tool implements a density segmentation, which is optimized for sibling comparisons, to find blocks of IBS types (Fig. 3, lower panel). Data are saved to a text file that can be viewed on the UCSC Genome Browser [17].

**Figure 4:**
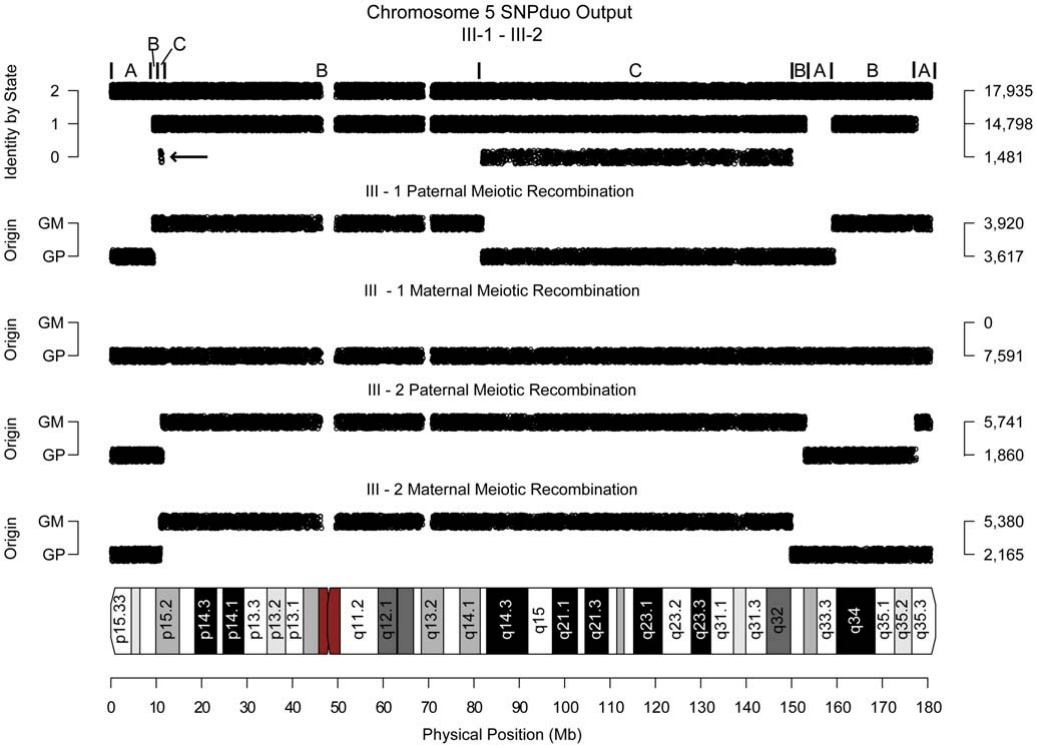
Meiotic recombination confirmed with synthetic family. Shown are SNPduo output and calculated grandparental origin for chromosome 5 of a synthetic family (based on Illumina HumanHap 550K platform) with respect to the two siblings. Panels displayed from top to bottom: (1) SNPduo output for the siblings on chromosome 5, (2) and (3) the recombination patterns for III-1's paternal and maternal chromosome 5, and (4) and (5) the recombination patterns for III-2's paternal and maternal chromosome 5. All grandparental origin change points corresponded to IBS track type change points in the SNPduo data. Abbreviations: GM: Grandmaternal origin. GP: Grandpaternal origin.

To confirm that changes in block type corresponded to meiotic crossovers we generated a synthetic data set (see Supplementary Information S1) for a family consisting of two siblings, their parents, and four grandparents (Fig. S1); the positions of all crossover events were known in the generation II parents and the generation III siblings. SNPduo and an IBS block type segmentation algorithm (see Supplementary Information S1) were applied to generation III siblings. Analysis of the synthetic pedigree confirmed that each IBS type change point corresponded to a meiotic crossover in either the mother or father for all chromosomes (Fig. 4). The figure shown was representative of the findings for all autosomes observed in the synthetic siblings. We observed similar findings with real data from multigenerational pedigrees (data not shown).

### SNPduo identifies six relationships in HapMap

**Figure 5:**
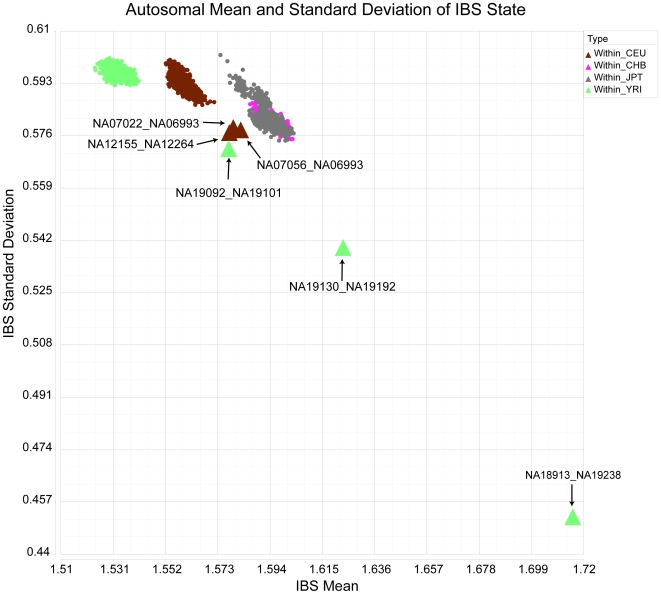
Unspecified relationships in phase 1 / 2 HapMap. Autosomal mean (x-axis) and standard deviation (y-axis) of IBS state data for the Affymetrix 500K platform visualized for all within group comparisons of a HapMap data set. Each data point corresponds to a pairwise comparison by a locally modified SNPduo. The six comparisons between related individuals are indicated as large triangles and annotated by name.

We analyzed nominally unrelated individuals from the HapMap project including individuals designated as parents from Yoruba (YRI; n = 60) and northern Europe (CEU; n = 60), and all individuals from China (CHB; n = 45) and Japan (JPT; n = 45). For this analysis we obtained the genotypes of all 270 phase 1 / 2 HapMap individuals assayed on the Affymetrix 500K platform (see Methods). We calculated the IBS state for all 210 unrelated individuals (n = 21,945 pairwise comparisons) across 22 autosomes. We next calculated the mean and standard deviation of the IBS states across all autosomes for each comparison. Related individuals were determined by plotting the mean versus standard deviation (using an approach similar to that described in the GRR software package [18]) and identifying outliers. The plot showed that the majority of pairwise comparisons had mean and standard deviation values that resulted in distinct clusters for pairwise comparisons within the YRI and CEU populations, and overlapping clusters for pairwise comparisons within the Asian (JPT and CHB) groups (Fig. 5). A total of 6 unexpectedly close relationships were discovered among the nominally unrelated individuals (Fig. 5).

(1) Yoruba individual NA18913 is the father in a trio while Yoruba individual NA19238 is the mother in a separate trio. However, the IBS analysis suggested that NA18913 and NA19238 shared a child/parent relationship. The SNPduo pairwise visualization for these individuals included IBS-1 type and IBS-2 type tracks overlaid (Fig. S2); this is a typical pattern for IBS-1 regions. We observed several regions with IBS-2 only, indicating some degree of historic relatedness between the putative mother (NA19238) and unknown father.

**Figure 6:**
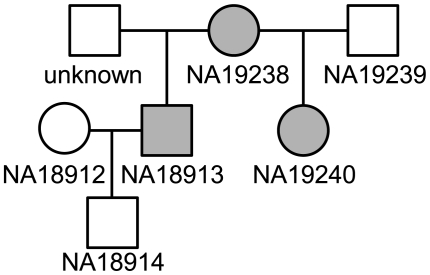
Inferred HapMap pedigree. Analysis of the mitochondrial SNPs between YRI individuals NA18913 and NA19238 showed a pattern of allele sharing consistent with a parent/child relationship. The two individuals shared the same mitochondrial haplotype, suggesting that NA19238 was the mother of NA18913, making NA18913 and NA19240 half-siblings. Shaded symbols indicated shared mitochondrial heritage.

We analyzed the allele sharing between the two individuals for non-redundant HapMap mitochondrial SNPs and identified a high degree of mitochondrial SNP sharing (180 shared alleles out of 181 mitochondrial SNP genotypes). Since mitochondrial SNPs are maternally inherited, this provided strongly suggestive, but not definitive, evidence for the model in Fig. 6 in which NA19238 is the mother of NA18913. Furthermore, while there are a limited number of mitochondrial haplotypes the greatest diversity is in the African population, increasing the significance of a shared haplotype in this group.

(2) The second relationship discovered was between Yoruba male individuals NA19130 and NA19192 (refer to pedigrees in Fig. S3 and SNPduo output in Fig. S4). There was evidence of segmental sharing of one allele on all chromosomes, including the X chromosome. This pattern was suggestive of a second degree relationship, for example half-sibling or avuncular. The X sharing indicated a shared female relative, since a common male ancestor would have passed on his Y chromosome to a male child. Two relationships were most plausible: half-sibling (Fig. S3a) and avuncular (Fig. S3b). In this arrangement the roles of uncle and nephew were interchangeable. The correct pedigree structure could not be identified from the data.

(3) The third unexpected relationship was between Yoruba individuals NA19092 and NA19101. There was evidence of segmental single allele sharing on about half the chromosomes of these two individuals (Fig. S5). The pair shared 154 HapMap mitochondrial SNPs IBS-2 with 25 IBS-0, and did not share X chromosome DNA. These data indicated that the pair did not share a female ancestor. The sharing pattern, as well as the mean and standard deviation compared to the unrelated Yoruba individuals, suggests a third degree relationship, such as first cousins (Fig. S6).

(4,5,6) The remaining relationships were between CEU individuals NA07022 and NA06993, NA12155 and NA12264, and NA07056 and NA06993. All of these individuals showed evidence of segmental single allele sharing throughout the genome. The pattern of allele sharing was similar to the previous case of putative first cousins, but on fewer chromosomes and with smaller shared regions. These data suggest the relationships between these individuals to be first cousins, once removed or an alternative relationship of a similar degree.

All of the relationships we described are consistent with those reported in a HapMap publication [19].

### Expected higher diversity in Yoruba compared to other HapMap groups

As stated previously, IBS-2 classifications correspond with IBS-2, -1, and -0 track types. IBS-1 classifications correspond with both IBS-1 and -0 track types. IBS-0 is the least common classification and corresponds to only the IBS-0 track type. Given that information content is inversely proportional to its frequency [20], the IBS-0 classified SNPs have the highest information content for determining unrelated individuals and amount of population variation since unrelated individuals demonstrate genome-wide IBS-0 track types. All pairwise comparisons of unrelated individuals within each HapMap population were performed using downloaded Affymetrix genotyping data. The number of IBS-0 classifications was counted for each autosome in each comparison. The X chromosome was excluded due to the confounding biallelic genotypes in hemizygous males. The Tukey Honest Significant Differences (Tukey HSD) is useful to define the statistical significance of differences for pairwise comparisons. We calculated Tukey HSD from the analysis of variance (ANOVA) of all of the within group comparisons using the R statistical programming language [21] (Fig. S7). The Tukey HSD allows for multiple comparisons while limiting the overall family-wise error rate (in this case to 5%), whereas multiple single tests would inflate the error rate. The Tukey HSD p-values are adjusted for multiple comparisons. Specifically, the Tukey HSD tested whether the within group IBS-0 counts differed between examined groups.

Several characteristics are apparent from the data. The within group YRI comparisons had a significantly greater number of IBS-0 counts (adjusted p-value<10^−7^) than the within group comparisons of CHB, CEU, and JPT. The JPT and CHB groups behaved similarly when compared to the YRI group, with both having between 95 and 105 fewer IBS-0 counts on average than the YRI group comparisons. The YRI group also demonstrated a greater number of IBS-0 counts than the CEU group, although the means differed by fewer IBS-0 counts (22 to 42 more IBS-0 calls in YRI than in CEU). The JPT and CHB groups showed no difference in the mean numbers of IBS-0 counts (adjusted p-value 0.62) in their respective within group comparisons. The CHB and JPT groups demonstrated similar differences in the mean number of IBS-0 counts compared with CEU with between 64 and 94 fewer IBS-0 calls (adjusted p-value<10^−7^).

By using the count of IBS-0 SNPs as a proxy for genotype diversity we concluded the YRI group had the greatest genetic diversity between the sampled “unrelated” individuals. CEU samples were more genetically similar than the YRI samples, but less genetically similar than the JPT or CHB samples. The JPT and CHB samplings appeared to be the most similar groups with respect to the genotyped markers, and they did not appear to be different from one another in terms of within group similarity. All of these findings were as expected based on previous HapMap analyses [19], [22].

### Differences in mitochondrial genome diversity between groups

The number of IBS-0 classified mitochondrial SNPs between all unrelated individuals within each HapMap group was tabulated, and the results were visualized by plotting the Tukey HSD values (Fig. S8). The data plotted were the square-root of the number of IBS-0 counts instead of the counts themselves. This was because in performing ANOVA one must assume that the variance within groups is equal, and especially that there is no mean-variance relationship. A simple box plot of the IBS-0 counts (data not shown) demonstrated increasing group variance with an increase in mean, an effect that the square root transformation removed. A log transformation was not used since the data contained counts of 0. The YRI group demonstrated a greater mitochondrial diversity than the CEU, CHB, or JPT groups (adjusted p-value<10^−7^). The CHB and CEU groups appeared to have no significant difference in the mean number of IBS-0 counts (adjusted p-value 0.59). However, the JPT group demonstrated less mitochondrial diversity as measured by the IBS-0 counts than either the CHB or CEU groups (adjusted p-value<10^−7^). This contrasts with the whole-genome result that the JPT and CHB group showed no difference in the number of IBS-0 counts between unrelated individuals for autosomal SNPs. A smaller amount of mitochondrial diversity with very similar genome diversity supports a small Japanese founder population.

These data indicated a hierarchy of mitochondrial SNP diversity within the sampled data. All groups had some individuals with shared mitochondrial haplotypes. The sampled YRI group had the greatest amount of mitochondrial diversity among unrelated individuals, while the JPT group had the lowest of amount of mitochondrial diversity in the sampled unrelated individuals. The diversity amongst the CEU and CHB groups was not significantly different.

### Analysis of a large autism data set

We obtained SNP data from 2,883 individuals genotyped on Affymetrix arrays in 721 multiplex autism families from the Autism Genetic Resource Exchange (AGRE). SNPduo++ analysis was performed to calculate the mean and standard deviation of IBS, the specified relationship, and a calculated relationship (unrelated, first degree [parent, child, or sibling], or identical sample) using a PLINK formatted ped and map file as input.

We highlight the usefulness of SNPduo++ in analyzing large datasets in two ways. First, we used SNPduo++ to find identical samples from those that were annotated in the pedigree file as non-identical. Twenty-seven identical sample pairs were identified (Supplementary Table S1). Twenty-six of these pairs were specified as siblings according to the pedigree (.ped) file, but calculated to be identical by SNPduo++. In these cases accompanying AGRE data confirmed that all of these pairs of siblings were actually identical twins (or higher order multiples). This highlights a weakness of the ped format. Without additional information it would be impossible to tell if these were in fact true identical siblings or if the samples were mislabeled.

For the remaining pair, a pairwise SNPduo++ calculation indicated that two samples were identical, but the pedigree file indicated that the genotype data were from unrelated individuals (Supplementary Table S1). This could occur if a sample is genotyped two or more times and was mislabeled. Less likely was the possibility that the individuals are in fact identical siblings adopted apart.

A second useful aspect of SNPduo++ is the identification of first-, second-, or third-degree relatedness in individuals who are annotated as unrelated. Twenty individuals specified as unrelated based on the pedigree file shared a first degree relationship (either parent/child or siblings; Supplementary Table S2). Many other individuals shared second or third-degree relationships (data not shown). Such findings allow one to move from the large-scale summary data back to the level of individual pedigrees. Extending and properly annotating pedigrees increases statistical power by increasing the number of families in the study and decreasing bias caused by not properly annotating those families.

We obtained a second set of 3,817 samples (which included reruns of some individuals) from AGRE genotyped on the Illumina 550K platform (n = 561,466 SNPs). These data were processed using command-line R scripts (see Methods) to calculate the mean and standard deviation of IBS state for all autosomes individually and summarily across all autosomes. Using these data several interesting phenomena were apparent.

One individual, AU009204, exhibited an unusual IBS sharing with his parents on chromosome 16. Compared to the mother, chromosome 16 consisted of a single IBS-2 block, but compared to the father chromosome 16 consisted of a single IBS-0 block. To rule out programming bugs, the identified individuals were directly exported from the Illumina BeadStudio software. Analysis by SNPtrio [23] confirmed a maternal uniparental heterodisomy of chromosome 16 in the child (Fig. S9). Given that this was a heterodisomy rather than a uniparental isodisomy, this event did not produce homozygosity on chromosome 16 in the child. Furthermore, all other chromosomes exhibited standard Mendelian inheritance, supporting a true UPD event and not a cell line artifact or software error.

Second, a mother, AU068501, exhibited unusual mean and standard deviation values on chromosome 18 when compared to her child. Examination in Illumina BeadStudio software revealed a mosaic monosomy of chromosome 18. There are two hallmarks that support this conclusion. The first is the decrease in intensity corresponding to reduced copynumber. The Illumina software provides a measure of intensity defined as the base 2 logarithm of the intensity of the individual versus a reference intensity (referred to as the Log R Ratio). The total intensity for a SNP is defined by the sum of both A allele and B allele probe intensities. The reference intensity is included in the software, and is derived from the average total intensity of that SNP for greater than 100 HapMap individuals. The decrease in Log R Ratio supports an interpretation of monosomy. The second hallmark is the homozygosity, evidenced in the Illumina data as B Allele Frequency. The B Allele Frequency is the fraction of total alleles that are a ‘B’ allele (which is derived from a series of transformations of coordinate intensity data). For example, with an AA genotype 0 out of 2 alleles are B, giving a B Allele Frequency of 0. For an AB genotype 1 of 2 alleles are B, giving a B Allele Frequency of 0.5. For a mosaic mixture of AB and A0, there are three alleles, and only one of them is a B. This gives a B Allele Frequency of approximately 0.33 (the same as an AAB trisomy genotype). An AB / B0 mosaic has two out of three alleles as a B genotype, giving a B Allele Frequency of approximately 0.67 (the same as an ABB trisomy genotype). For this individual the coordinate drop in Log R Ratio and splitting of the B Allele Frequency panel into tracks at 0, 0.33, 0.67, and 1 indicate a mosaic monosomy (Fig. S10). Without the intensity information it would be impossible to determine if it was a mosaic monosomy or a trisomy.

A number of samples appeared to have been genotyped twice in this dataset, presumably due to low genotype call rates evident during the first run. The rerun set had accepted genotyping rates and less noise, but IBS analysis revealed the rerun samples were mislabeled and did not agree with the specified relationships in the pedigree information. We use SNPduo++ IBS analysis to reconstruct pedigrees for these individuals (Fig. S11). The sample which demonstrated maternal uniparental heterodisomy, AU09204, was included in the mislabeled group. The nuclear family was reconstructed using SNPduo data (Fig. S11, family B), but we cannot determine which true individual ID the data belongs to, i.e. whether AU09204 from the rerun group corresponds to the true identity of AU09204. In large datasets, particularly studies with thousands of individuals, sample mislabeling is routine. These findings highlight the need for applying quality control measures to data after genotyping to reduce the impact these types of errors could have on data analysis.

The number of unlinked markers required to discern unrelated individuals from those with closer relationships is on the order of a few hundred markers (Roberson and Pevsner, in preparation). However, randomly sampling markers from larger data pools would fail to identify smaller scale changes, such as a single chromosome UPD or a segmental copy number alteration.

## Discussion

SNPs are useful markers for many types of genetics and population-based studies. A challenge of SNP data analysis is to identify shared chromosomal segments in both closely and distantly related individuals. In this work we present SNPduo, a tool that identifies and visualizes shared loci.

SNPduo is a useful analysis tool in several different situations. (1) Large-scale SNP association studies can involve hundreds or thousands of presumably unrelated samples. By plotting mean and standard deviation values generated by SNPduo++, unexpected first-, second-, and third-degree relationships can be revealed. The --conflicting switch for SNPduo++ can also identify identical samples and first degree relationships. (2) PLINK is a popular tool for running association studies and many users generate a file containing IBD probabilities and IBS counts using the --genome option. The output file can be fed into SNPduo++ directly without having to rerun the entire analysis, facilitating the examination of population structure and cryptic relationships by calculating the mean and standard deviation of IBS for each comparison. In this scenario, in which the PLINK output does not include pedigree information, the specified relationships cannot be determined. (3) The web-based version of SNPduo is useful for smaller data sets, particularly within families. Using the web based version of SNPduo in family studies can help reveal shared ancestry in parents, the transmission of alleles through generations (such as grandfather to grandson), uniparental inheritance, location of meiotic crossovers between siblings, and the parental chromosome that is affected in hemizygous deletions.

Two previously described software tools are often used to determine relationships between individuals, RELPAIR and GRR [18], [24]. RELPAIR is written in Fortran 77 and based on the source code the initial limit of SNPs is 10,000. Current studies use hundreds of thousands to millions of SNPs per individual, far more than the suggested maximum. RELPAIR also requires a priori knowledge of the allele frequencies for each marker to be listed in the locus file, which may not be easily derivable for all marker sets in all populations, and may require a certain amount of estimation. GRR is another widely used option. The GRR documentation suggests using at least 200 markers, but is more reliable with 300 or more. Modern SNP studies use several thousand times more markers, which may require attempting to pare a dataset down to a few hundred markers selected at random from the genome.

The Merlin and PLINK software packages can perform pairwise IBD probability (*P*_IBD_) estimations of the probability that two individuals share 0, 1, or 2 alleles IBD. The PLINK output is a per individual *P*_IBD_ estimation, along with a summary of IBS counts for each IBS type across all chromosomes. This output can be imported into SNPduo++ to generate mean and standard deviation of IBS calculations. Merlin is capable of generating single-point *P*_IBD_ estimations for many markers, but all such probability calculations rely on accurate assumptions about allele frequencies in a test population. The use of summary IBS in SNPduo++ and single-point IBS calculations in SNPduo avoids these population assumptions.

In conclusion, SNPduo offers several advantages in dataset analysis. It is designed to work with datasets that contain hundreds of thousands to millions of SNPs. The SNPduo++ source code is freely available for local download and implementation on a variety of computer architectures. The scripts for the web based SNPduo are also available for local implementation, though they are not portable to architectures other than Linux at this time. There are many available tools for analyzing SNP data in populations (e.g. RELPAIR, GRR, Merlin, PLINK). The SNPduo tools are designed to complement other available tools for large-scale data analysis without replacing their functionality, as well as provide visualization tools for smaller scale analysis. The web based SNPduo allows for researchers who are not programmers to directly export data from standard genotyping software (e.g. from Affymetrix, Illumina, or HapMap bulk data downloads), upload it to a website, and obtain both tabular and visual output.

The tracks visualized on the SNPduo web output have low noise given accurate genotype calls. The simple density segmenting algorithm for finding blocks of IBS type (see Supplementary Information S1) relies on this high signal to noise ratio; it is most well-suited to sibling data where there are a variety of discrete regions of different track types. More sophisticated models, such as a Hidden Markov Model or Circular Binary Segmentation, could be implemented as well. However, the density algorithm is faster and less computationally intensive for high-density data than model-based alternatives.

The SNPduo web tool is useful for visualizing meiotic crossover points that occurred in siblings. Recent publications examining recombination use two [25], [26] or three [27] generations of a family to discover the location of a recombination event. SNPduo uses a single generation of siblings, which is more practically attainable than two or three generations. However, there are limitations in using only one generation. SNPduo can visualize the position of crossovers in siblings, but is unable to phase which sibling's chromosome the crossover occurred on, or to report which parent the crossover was derived from. Therefore it is useful in determining the position of crossovers, but not in determining the interference distance between crossovers on a chromosome or sex-specific recombination rates, since both require allele phasing.

## Methods

### SNPduo: web implementation

The SNPduo web tool consists of scripts that manipulate input into the proper format, determine the single-point IBS state of each SNP in a comparison, and generate the SNPduo output, which includes web tables, downloadable files, and images. The tool is web-accessible at http://pevsnerlab.kennedykrieger.org/SNPduo.

The source scripts are available for download from the SNPduo website. Local implementation of the SNPduo web tool requires an appropriate operating system, required software packages, and some code customization (for file and directory locations). The CGI script that parses input data, triggers system commands, performs text manipulations, converts image formats, and outputs the results is written in Perl. SNP level analysis is performed using R functions interfaced with compiled C code; postscript images are created using R as well. IBS classification is performed according to the SNPduo schema (Table 1). All of the R and C code used for processing IBS analyses were custom generated for this tool and not ported from existing packages.

**Table 1:** SNPduo IBS Calling Schema.

Genotype 1	Genotype 2	Identity-by-State
AA	AA	2
AA	AB	1
AA	BB	0
AA	No Call	NA
AB	AA	1
AB	AB	2
AB	BB	1
AB	No Call	NA
BB	AA	0
BB	AB	1
BB	BB	2
BB	No Call	NA
No Call	AA	NA
No Call	AB	NA
No Call	BB	NA
No Call	No Call	NA

Genotype 1 and 2 are the genotypes of the two individuals being compared. Genotypes are shown in the binary A/B genotype convention. Uncalled alleles in either individual are uninformative of IBS and are not plotted. Abbreviation: NA, not applicable.

Data are uploaded via a web form. The upload form gives customizable options including how columns are delimited, the data platform, the columns of individual's data to compare, the chromosome(s) of interest, output page size, the genome build the data are based on, and whether to generate PNG images and Postscript files of the output. Analysis can be run on two individuals, or a batch of all possible comparisons can be performed on a group of columns. It should be noted that the number of operations increases nearly exponentially with each individual added to batch mode operations. Comparisons that include image generation are best suited for comparisons between up to five to ten individuals due to the length of time required for image creation. A single chromosome, a subset of chromosomes, or all included chromosomes can be examined in separate plots or in a combined genome plot. Data are delivered on an output page that shows a PNG, if created, and provides download links to all of the generated files. These include an output file with the chromosome, position, genotypes, and IBS classification of every SNP, a BED file of shared segments for display on the UCSC Genome Browser [17] and a publication quality postscript of the data visualization, if selected. Included on the output page are tables that show summary data for the genotype distribution and IBS class distribution for the analyzed data.

For larger datasets (tens of individuals) where all possible pairwise comparisons are desired without image generation, the tabulate option, which is more optimized for speed, can be used. The results are two summary files. The first lists the individual, the chromosome, and the count of each genotype class. The second file lists the two individuals compared, the chromosome, and the count of each IBS class. The data are also displayed on the result page as two separate tables.

For datasets with tens of individuals segmenting may also be accomplished in a reasonable amount of time if no images are generated (by deselecting all image generation options during batch mode). For example, segmenting all pairwise comparisons of 25 individuals (n = 300) for ∼50,000 markers on one chromosome required approximately 7 minutes on a server with 3.0 GHz Xeon processors, and did not trigger a browser timeout.

### SNPduo++: command-line implementation

SNP datasets with high-density, genome-wide coverage for many individuals (hundreds to thousands) are intractable via a web interface using interpreted code. The time spent uploading the file, generating images, and performing computations would cause the connection to timeout. However, optimized and compiled code run locally is well-suited suited to this task.

A version of the SNPduo algorithm was written in C++ to make the SNPduo++ program. The command-line program accepts either pedigree format files, including a “.ped” and a “.map” file, or a transposed pedigree format, including a .tped and a .tfam file. These formats were chosen because they are the same input formats used by the popular analysis tool PLINK. A ped file requires seven columns containing family ID, individual ID, father ID, mother ID, sex (integer format), and phenotype (integer format), followed by individual alleles of each genotype. The map files include chromosome, RSID, genetic distance (optional), and physical position. The transposed format puts family information in a “.tfam” file, while annotation and genotypes go in a “.tped” file. The tfam file consists of the first seven columns found in a ped file. The tped file contains the columns of a map file, followed by genotypes.

When running the command-line version of SNPduo++, an input source is specified along with one or more analysis or recoding options. Data are read in from one of the supported file formats into memory for analysis. The selected analysis options are performed, and output is written to disk in files. Since the command-line version is written in C++ it is highly portable, and should run everywhere a C++ compiler works, i.e. Windows, Mac, and Linux platforms in both 32-bit and 64-bit environments. It should be noted that SNPduo++ seeks an end of line character (\n) to delimit new lines, ignoring carriage returns (\r). Mac users may have to convert end of line carriage returns to newline characters for the tool to parse files correctly.

Memory in excess of 4 Gb can be used if the code is compiled in a 64-bit environment. However, genotypes are stored in specialized vectors to reduce memory usage. As a result, a file greater than 4 Gb on disk can be represented at ∼500 Mb in memory. A recoding switch, --recode --webduo, will generate a text file compatible with Custom Format of the web implementation of SNPduo to explore datasets further. For SNPduo++ (as for PLINK), the genotypes of a given SNP are specified by any two alphanumeric characters, e.g. A/B format, 1/2 format, A/C/G/T format, etc. The --webduo option converts the read alleles to the A/B format to allow analysis of the genotype data with the web-based SNPduo that requires A/B coded genotypes.

### Benchmarking SNPduo++ and PLINK

We benchmarked the performance of PLINK (v1.03) and SNPduo++ (v1.01b) to compare functionality and speed. Tests were performed on a server with four 3.00 GHz Xeon processors and 6 Gb of RAM running Red Hat Linux kernel 2.6.9–78.0.1.ELsmp. Both programs were compiled from source code using make (v3.80) and g++ (v3.4.6). Compile flags used optimization level three, and march / mtune with the parameter “nocona”. Benchmarking data was the AGRE Affymetrix autism family dataset analyzed elsewhere in this paper, in the standard ped / map file formats. Each program was tested for the ability to analyze all possible comparisons of individuals in the dataset (n = 4,154,403). The PLINK --genome finished in 39 hours 25 minutes. The performance of SNPduo++ to count IBS states between individuals was benchmarked using the --counts switch. The analysis completed in 12 hours 5 minutes, an approximately 70% shorter run time. Since SNPduo++ does not perform marker sorting, IBD calculations, marker missingness calculations, or tests of Hardy-Weinberg equilibrium it can perform IBS counting operations much more quickly. As such, SNPduo++ is not a replacement for PLINK functionality, but instead is software optimized for specific SNP calculations.

### Analysis of HapMap data

The genotypes of 270 HapMap individuals genotyped on the Affymetrix 500K platform using the BRLMM algorithm were downloaded from the Affymetrix website (http://www.affymetrix.com/support/technical/sample_data/500k_hapmap_genotype_data.affx, accessed March 26, 2008). The genotypes were coded into a numeric format and stored into a local MySQL database. The SNPduo R code was used to analyze the data. The pedigree data from HapMap were used to select all individuals with both parents designated as 0, i.e. unrelated individuals (n = 210). On a chromosome by chromosome basis these individual's genotypes were queried from the database to be used as input genotypes, and the counts of each IBS class for each comparison were tallied. In total this involved 21,945 inter-individual comparisons for 23 chromosomes.

The most closely related individuals were defined by calculating the mean and standard deviation of IBS state across all autosomes for each comparison. The mean and standard deviation data were visualized in the Partek Genomics Suite software (Partek Incorporated, St. Louis, MO). Data were imported such that the individuals compared were text labels, the comparison group was a categorical factor, and the mean and standard deviation were double precision response variables. Outliers were visualized by plotting the mean versus standard deviation for each comparison. The outliers were then individually selected and further studied to define the relationship.

### Analysis of Illumina autism data

Illumina 550K autism family data were exported from BeadStudio, including only chromosome, physical position, and genotypes. Data were numerically coded into a 0, 1, 2, 3 convention representing NC, AA, AB, and BB, respectively. Converted data were stored in a MySQL database.

Counts of each IBS state were determined for each chromosome. The counts were added for all autosomes, and used to calculate the autosomal mean and standard deviation of IBS. The per-chromosome counts were visualized and examined for outliers (reported in results) using the R Statistical Programming Language and Partek Genomics Suite.

### Availability and requirements

SNPduo++ is platform independent; the SNPduo webtool requires Linux for local installation, and the webtool is accessible from any browser. SNPduo was programmed in Perl, R, C/C++, PHP, and is available without restrictions.

## Supporting Information

Supporting Information S1 Methods for identifying meiotic crossovers, synthetic data generation, and a segmenting algorithm.
(0.04 MB DOC)

Figure S1 Pedigree representing synthetic data used for meiotic recombination analysis. Shown is a standard three generation pedigree, which includes two sets of grandparents, two parents, and two children (male and female). Individuals I-1, I-2, I-3, and I-4 were generated as new synthetic individuals. II-1 was generated as the child of I-1 and I-2; II-2 was generated as the child of I-3 and I-4. III-1 and III-2 were generated as the children of II-1 and II-2.
(0.17 MB DOC)

Figure S2 The SNPduo chromosome 17 comparison for Yoruba HapMap individuals NA19238 and NA18913 based on Affymetrix genotyping data. The single IBS-0 call was consistent with a low genotyping error rate, and supported the hypothesis that the entire chromosome was actually an IBS-1 type track having IBS-2 and IBS-1 calls. The plot contained an unusual, small segment of IBS-2 SNPs (black arrow). The area did not correspond to homozygosity, ruling out autozygosity (shared homozygosity by descent). It represents some degree of relatedness between the parents of this pedigree.
(0.88 MB DOC)

Figure S3 Two possible pedigrees for the degree of relationship shared between NA19192 and NA19130. NA19130 and NA19192 were close relatives who shared all HapMap mitochondrial SNPs. Two pedigrees could have likely explained this relationship. Panel A demonstrates a half-sibling relationship, where they have the same mother but different fathers. Panel B shows the two in an avuncular relationship. The position of the NA19192 pedigree could have been swapped with that of NA19130 in this case. Shaded symbols indicated individuals with shared mitochondrial inheritance.
(0.34 MB DOC)

Figure S4 SNPduo output demonstrating variable allele sharing between HapMap individuals NA19130 and NA19192 (genotyped on the Affymetrix 500K platform). Segmental sharing of one allele is observed for chromosome 14. An area of IBS-2 can be seen near the distal end of the q-arm.
(0.82 MB DOC)

Figure S5 Variable allele sharing from no shared alleles (panel A) to segmental sharing of one allele (panel B). SNPduo output is shown for YRI HapMap individuals NA19092 and NA19101 genotyped on the Affymetrix 500K platform. (A) Chromosome 2 is an example of the two individuals sharing no inherited DNA for the entire length of a chromosome. (B) Chromosome 1 demonstrated variable sharing of one allele across an entire chromosome as well as regions of no shared alleles.
(0.93 MB DOC)

Figure S6 Inferred pedigrees for HapMap NA19092 and NA19101. Shown are two possible pedigree configurations that would explain the relationship between NA19093 and NA19101 if they are first cousins. Panels A and B are two configurations for a first cousin relationship. Both configurations took into account the lack of a shared mitochondrial haplotype. In this figure diamonds indicate individuals where the sex of the individual is irrelevant in the context of the mitochondrial inheritance pattern. Shaded symbols had common mitochondrial inheritance. A half-shaded symbol may or may not have shared mitochondrial lineage based on ambiguous parental assignment in the previous generation.
(0.20 MB DOC)

Figure S7 A Tukey Honest Simple Differences boxplot showed differences in the number of autosomal IBS-0 counts for within group comparisons of the four original HapMap groups. The Tukey Honest Simple Differences were plotted for the difference by group in the number of IBS-0 classifications for all within-group comparisons. The YRI group demonstrated more diversity than CEU, CHB, or JPT groups as determined by the number of IBS-0 counts between unrelated individuals in that population. The CHB and JPT groups demonstrated the lowest diversity, but with no difference in the mean diversity between them.
(0.51 MB DOC)

Figure S8 Tukey HSD boxplot of the number of IBS-0 counts for within-group comparisons of mitochondrial SNPs for the four original HapMap groups. The Tukey Honest Simple Differences were calculated for the square-root of the mitochondrial IBS-0 count amongst all unrelated individuals. Mitochondrial genotypes were downloaded from the HapMap project website. The YRI group showed an increased amount of SNP diversity as compared to the CEU, CHB, and JPT groups. The JPT group demonstrated the lowest amount of mitochondrial SNP diversity between unrelated individuals. The CEU and CHB groups showed greater diversity than the JPT group and less than the YRI group, but no difference in the mean number of IBS-0 counts between them.
(0.49 MB DOC)

Figure S9 Identification of UPD 16 in the AGRE autism dataset. Shown is SNPtrio output for AU009204 (rerun) and AU009201 (rerun) compared with the detected parents on chromosome 16 (Illumina data). While AU09201 demonstrates routine biparental inheritance for the entire chromosome, AU09204 demonstrates maternal uniparental heterodisomy for the entire chromosome. Both individuals show a nominal Log R Ratio, indicating that the pattern cannot be explained by copy number variation.
(0.65 MB DOC)

Figure S10 Inferred pedigrees for AGRE mislabeled samples: identification of mosaic monosomy18 in an AGRE proband. Chromosome 18 Illumina marker data are shown for AU068501. The top panel contains the B Allele Frequency data (genotype data), and the bottom panel contains the Log R Ratio (intensity data). The entire chromosome exhibits a dip in Log R Ratio, indicating a loss of DNA. If the sample had 100% monosomy then the corresponding B Allele Frequency would split between 1 and 0. However, the upper panel is split into four tracks, indicating a mosaic monosomy of chromosome 18.
(0.98 MB DOC)

Figure S11 We reconstructed pedigrees from AGRE autism data (Illumina platform). For apparently mislabeled samples we used SNPduo to derive mean and standard deviation of IBS values, then inferred relationships of individuals. The data demonstrate that the rerun samples do not correspond to the original sample ID. However, the true sample ID in each case is unknown.
(0.56 MB TIF)

Table S1 The samples shown are those identified as identical to each other. These are mostly identical siblings, with the exception of family CEPH, individual 1 and family 12144, individual 12144. These were identical samples labeled as independent individuals. Abbreviations: FID = Family ID. IID = Individual ID. SD = Standard Deviation. Specified = Relationships from ped file. Calculated = Determined from Mean / SD.
(0.06 MB DOC)

Table S2 Listed in this table are individuals the pedigree file specified as being unrelated, but appear to be first degree relatives by IBS analysis. While differentiating between different degrees of relatives can at times be difficult due to genotyping errors, missing calls, and varying population allele frequencies, it is almost always trivial to discriminate between unrelated individuals and first degree relatives in any large dataset. Abbreviations: FID = Family ID. IID = Individual ID. SD = Standard deviation. Specified = Relationship specified in pedigree information. Calculated = Relationship calculated from the Mean and SD of IBS.
(0.05 MB DOC)

Table S3 Crossover detection schema. Four individuals are required: a child, a parent, and the grandparents on the chosen parent's side. Only homozygous child SNPs that correspond to heterozygous parental SNPs can be informative. Each of the informative SNP types can conclusively link one allele to a particular grandparent. Abbreviations: GP: Grandpaternal, GM: Grandmaternal, PA: Parent, CH: child, GP-T: Grandpaternal-type, GM-T: Grandmaternal-type.
(0.04 MB DOC)
