# Supplementary material for: Visualization of Shared Genomic Regions and Meiotic Recombination in High-Density SNP Data
Source: PLoS One. 2009 Aug 21;4(8):e6711. doi: 10.1371/journal.pone.0006711 (PMC2725774; doi:10.1371/journal.pone.0006711)
Supplement: Supporting Information S1 — Methods for identifying meiotic crossovers, synthetic data generation, and a segmenting algorithm. (0.04 MB DOC) [file pone.0006711.s001.doc]

**Supporting Information S1**

**Identifying meiotic crossovers in three generation pedigrees**

The grandparental origin of a SNP allele can be determined for informative SNPs. A visual approach that created plots showing grandparental origin was adapted from previously published methods [1]. A schema was used to determine which SNPs were informative, and of those which grandparent the alleles were derived **(Supplementary Table 3)**. An informative SNP must be homozygous in the child and heterozygous in the parent; it also must have grandparental genotypes that are differentiable with respect to which grandparent passed the A allele and which passed the B allele to the parent. High-density SNP data are well-suited to this task, since within a given family informative SNPs may represent only a small fraction of the total number of SNPs. Plots of grandparental allele origins were generated in R.

**Synthetic data generation**

Synthetic data for the Illumina HumanHap550K genotyping platform were generated in R using reference genotyping data. Sex could be selected manually or automatically with equal probability. For females the true copy number for every SNP was set to 2; males had a copy number of 1 for the X chromosome, and a copy number of 2 for autosomes. Genotypes and Log_2_ (R_subject_/R_reference_) ratios (referred to as Log R Ratios) were selected for every SNP from the reference genotype data, excluding SNPs in the pseudoautosomal regions, mitochondria, and Y chromosome.

Genotypes were selected for each SNP based on a weighted probability of drawing that genotype from the given copy number’s genotype distribution. Let *G* be the set of possible genotypes in the form of 0, 1, 2, or 3 representing No Call, AA, AB, and BB genotypes; let *C* be the copy number of 1 or 2. The probability of selecting *G_i_* from *G* given the copy number *C* is equal to the count of the number of times *G_i_* occurs in the set of training genotypes for *C* divided by the total number of genotypes for *C*. The genotype probabilities were generated from the reference data containing 566,619 SNPs from 41 female X chromosomes for copy number 2 and 469,914 SNPs from 34 male X chromosomes for copy number 1. Log R Ratios for each SNP were drawn at random with replacement from the set of Log R Ratios for a given copy number. After generating the Log R Ratios and genotypes, haplotypes were generated for each parental allele of the synthetic individual.

For each SNP an allele was assigned to the maternal and paternal haplotypes at random. Any “No Call” alleles were first randomly assigned a “true” genotype from a choice of AA, AB, or BB. The creation of haplotypes was the last step in creating a synthetic individual.

Synthetic children were then generated from synthetic parents. The child’s sex could be specified or generated automatically with equal likelihood for either sex. For each parent gametes were generated. First the location and number of crossover points were determined for each chromosome. A maximum of two crossovers were possible, and the exact number was selected based on the length of the chromosome arm. Let *L* be the length of a given chromosome arm. For *L* less than 60 Mb only one crossover site is selected. For *L* between 60 Mb and 80 Mb there is an equal probability of selecting either one or two crossovers. For *L* greater than 80 Mb the probabilities of selecting one or two crossovers are 0.35 and 0.65, respectively.

Let *B* be a vector of the available 1 Mb bins on a chromosome arm available for selecting a crossover. Let *s* and *e* be vectors of the genomic start and end positions for the corresponding bin *B*. Also let *w* be a vector of weights in Morgans per Mb for each bin in *B* corresponding to the probability of a crossover occurring in a bin. The probability *w_i_* of choosing a 1 Mb block *B_i_* is weighted using the probability for a crossover as determined from sex-specific recombination rates [2] in Morgans per Mb exported from the UCSC Genome Browser [3]. Vector *b* was an integer vector of all positions from the start to end positions *s_i_* and *e_i_* of the bin *B_i_*. The exact position of the crossover was selected from *b* randomly. An interference range of 20 Mb was simulated by removing up to 20 bins on either side of the crossover from the set of possible blocks. This method did not select for crossovers to occur specifically at a SNP, since in reality a crossover does not often conveniently occur at the exact position of a SNP, or necessarily in a region of high SNP density. Once the positions of the crossovers were determined in an individual, two recombinant gametes were generated.

First two recombinant gamete vectors were generated representing a paternal recombinant and a maternal recombinant gamete. On a SNP by SNP basis the vectors were populated with alleles. In regions where the SNPs should be the same as the non-recombinant gamete alleles, the allele for that position was taken from the same parental haplotype. In regions of crossover alleles were taken from the opposite parental haplotype. The two new haplotypes represented the maternal and paternal recombinant gametes. This gives a total of four possible gametes for each chromosome: maternal non-recombinant, maternal recombinant, paternal recombinant, and paternal non-recombinant. For each chromosome one of the four gametes was selected at random. The X chromosome was an exception to this rule in synthetic fathers. In fathers the maternal non-recombinant haplotype was always selected if the child was female and a null chromosome was selected if the child was male.

The child’s genotype was assembled from the transmitted gametes. Genotyping error was omitted for the sake of simplicity. A synthetic child then had a copy number assigned for the SNPs in his or her genome. Females were assigned copy number 2 for all SNPs, and males were assigned copy number 2 for autosomal SNPs and copy number 1 for X chromosome SNPs. Log R Ratios for each SNP were drawn from the reference distributions appropriate for the copy number as described for new synthetic individuals.

The end result was a synthetic child with genotypes, Log R Ratios, and parental haplotypes. Synthetic children could then be used to generate more synthetic children, which made it possible to generate any conceivable pedigree construction. This synthetic data generation method was focused on producing known meioses, and as such the random draw of genotypes for founders results in over-representation of heterozygote genotypes, making it unsuitable for generating synthetic data for experiments that rely on appropriate heterozygote frequencies.

**Segmenting Algorithm**

Many methods exist for segmenting data. However, model based methods such as Circular Binary Segmentation and Hidden Markov Models often have substantial computational cost associated with them. SNPduo uses a simple, index-based method to decrease computational time.

Blocks considered IBS-0 type blocks contain IBS-0 SNP state calls accompanied by IBS-1 and IBS-2 calls. IBS-1 blocks overlap IBS-1 calls and IBS-2 calls, but not with IBS-0 calls. IBS-2 blocks consist only of IBS-2 SNP calls, not IBS-1 or IBS-0. This allows for the possibility of iterative processing. Clusters of IBS-0 SNPs will indicate an IBS-0 block. Once an IBS-0 block is identified, all of the SNPs in that block (IBS-0, IBS-1, and IBS-2) can be removed. Next all of the IBS-1 SNPs are examined to find IBS-1 blocks. IBS-1 blocks are then removed, leaving IBS-2 blocks. A fourth pattern, consisting of IBS-0 and IBS-2 is seen when comparing two individuals that have non-identical hemizygous deletions, such as comparing male X chromosomes. To find this block type all IBS-0 blocks are tested for IBS-1 SNPs. If the IBS-1 SNPs are very low density, the region is marked as an aberration.

The segmenting algorithm is highly efficient when using high-density SNP data. The segmenting algorithm works one chromosome at a time on a vector of IBS states that have been sorted by physical position. Initially the size of the vector and the count of each IBS state are determined. These values are used to calculate the maximum index distance between SNPs of the same state that can be considered part of a block and the minimum number of SNPs to determine a block. Thus we efficiently exclude outlier SNPs from blocks. Maximum index distance between SNPs is calculated as 1.75%, 1.5%, or 0.5% of the total number of SNPs for IBS-0, IBS-1, and IBS-2 types, respectively. A maximum index of five is substituted for values less than five to avoid aberrant single SNP blocks. These settings have proven appropriate for several SNP platforms of varying SNP data (including Affymetrix 6.0, and Illumina 550K and 1M platforms, and high density HapMap data).

The minimum number of SNPs required to define a block is calculated as 0.75% of the count for that type or five, whichever is greater. A hard limit of five was determined empirically to be the minimum number of SNPs required to avoid creating extensive false block structures due to random genotyping error.

To process any type of block, the input IBS state vector is scanned for that IBS type, and a second vector, a vector of indexes, is created that contains the index of every instance of that IBS type in the input. The index vector is processed to determine the distance between each index (a vector of differences). Indexes within the maximum allowed distance of each other for that type are binned into block. Blocks are broken when the distance between two vector members is greater than the maximum allowable for the type, or the end of the distance vector is reach. Blocks containing at least the minimum number of SNPs are kept as true blocks. The entire process is iterated again for the next SNP type until all three types are processed. After each iteration SNPs defined as being within a block are removed to prevent blocks from overlapping.

After all blocks have been determined, IBS-0 blocks are analyzed to determine if they are in fact IBS-0 blocks or aberrations. The number of IBS-1 SNPs in an IBS-0 block are counted. If IBS-1 SNPs account for less than 10% of the total number of SNPs in the region, it is redefined as an aberration. While generally robust, small blocks of only a few SNPs (less than 20 or so), or blocks that cover an area of sparse SNP density occasionally don’t have any IBS1 calls by chance. These small aberration type blocks should be confirmed by manual inspection.

**Supplementary References**

1. Cheung VG, Burdick JT, Hirschmann D, Morley M (2007) Polymorphic Variation in Human Meiotic Recombination. American Journal of Human Genetics 80: 526-530.

2. Kong A, Gudbjartsson D, Sainz J, Jonsdottir GM, Gudjonsson SA, et al. (2002) A high-resolution recombination map of the human genome. Nat Genet 31: 241-247.

3. Kuhn RM, Karolchik D, Zweig AS, Wang T, Smith KE, et al. (2009) The UCSC Genome Browser Database: update 2009. Nucl Acids Res 37: D755-761.
