# Supplementary figures and images for: Visualization of Shared Genomic Regions and Meiotic Recombination in High-Density SNP Data
Source: PLoS One. 2009 Aug 21;4(8):e6711. doi: 10.1371/journal.pone.0006711 (PMC2725774; doi:10.1371/journal.pone.0006711)

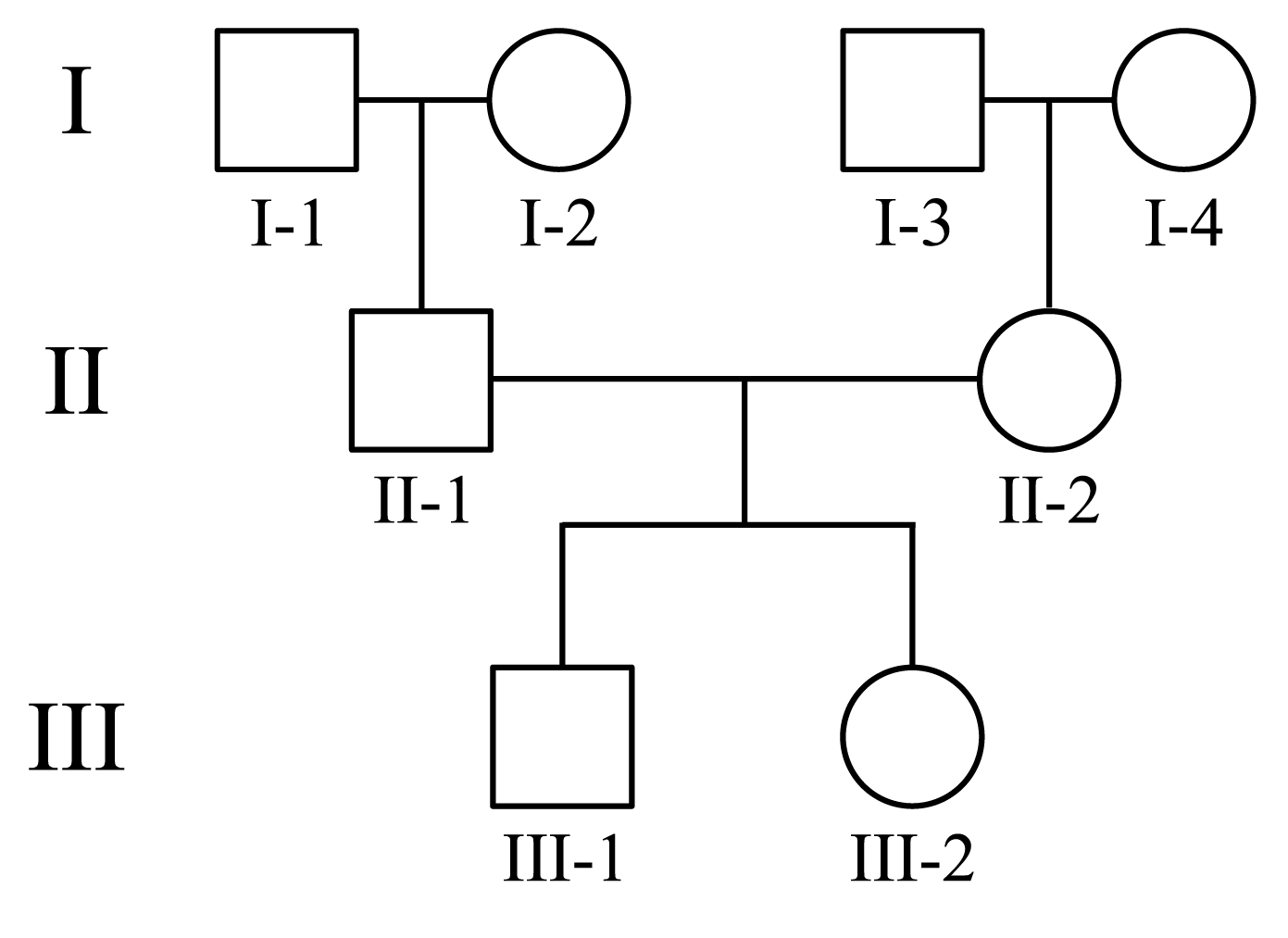

Supplement: Figure S1 — Pedigree representing synthetic data used for meiotic recombination analysis. Shown is a standard three generation pedigree, which includes two sets of grandparents, two parents, and two children (male and female). Individuals I-1, I-2, I-3, and I-4 were generated as new synthetic individuals. II-1 was generated as the child of I-1 and I-2; II-2 was generated as the child of I-3 and I-4. III-1 and III-2 were generated as the children of II-1 and II-2. (0.17 MB DOC) [file pone.0006711.s002.tif]

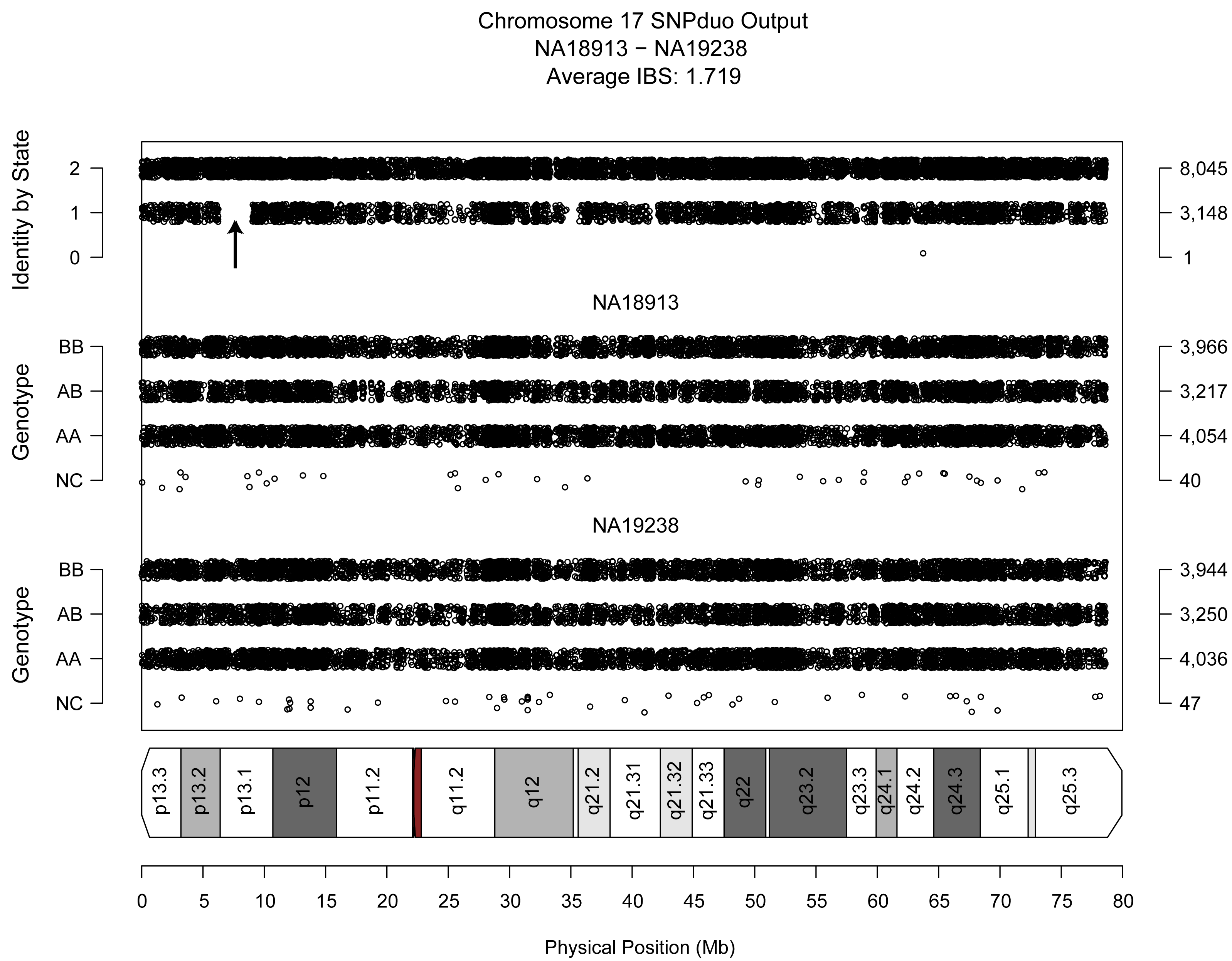

Supplement: Figure S2 — The SNPduo chromosome 17 comparison for Yoruba HapMap individuals NA19238 and NA18913 based on Affymetrix genotyping data. The single IBS-0 call was consistent with a low genotyping error rate, and supported the hypothesis that the entire chromosome was actually an IBS-1 type track having IBS-2 and IBS-1 calls. The plot contained an unusual, small segment of IBS-2 SNPs (black arrow). The area did not correspond to homozygosity, ruling out autozygosity (shared homozygosity by descent). It represents some degree of relatedness between the parents of this pedigree. (0.88 MB DOC) [file pone.0006711.s003.tif]

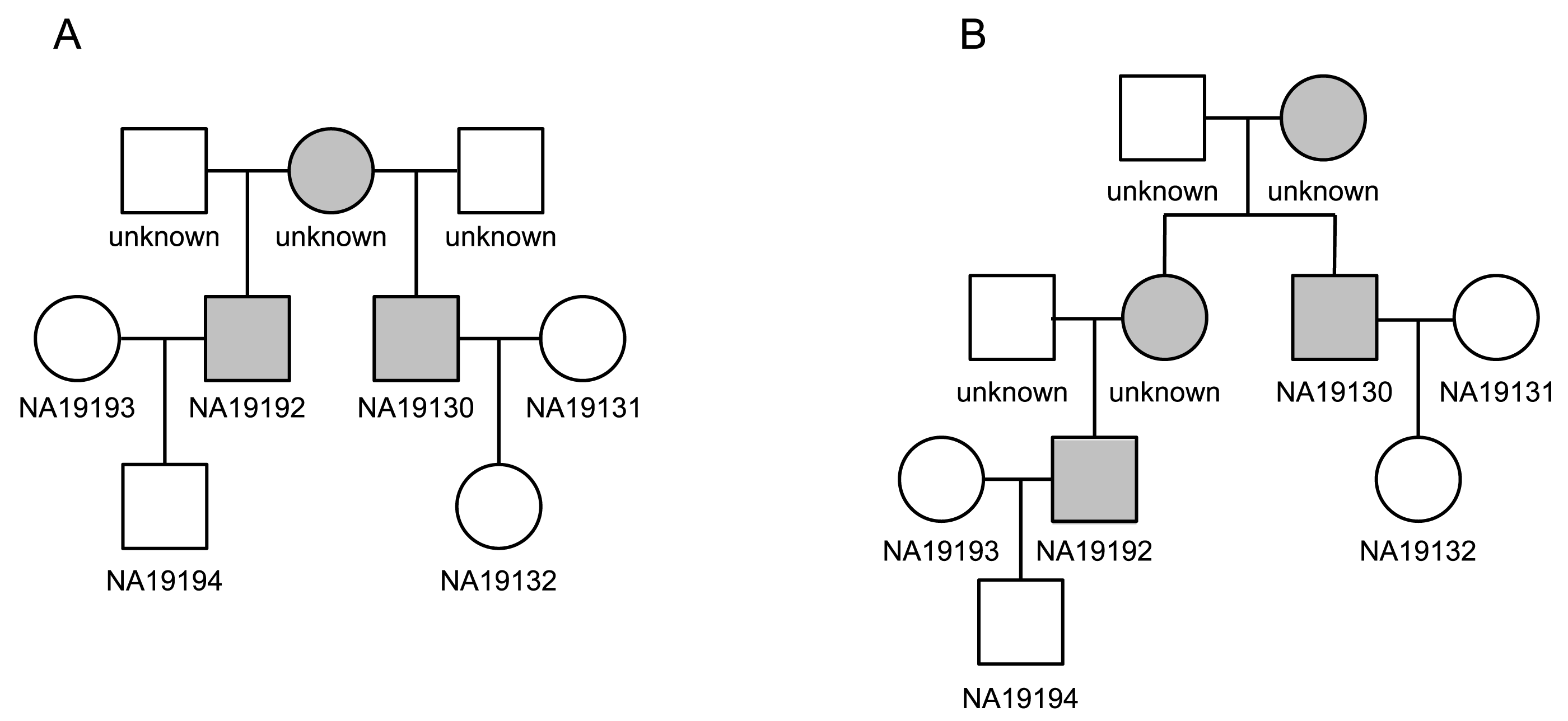

Supplement: Figure S3 — Two possible pedigrees for the degree of relationship shared between NA19192 and NA19130. NA19130 and NA19192 were close relatives who shared all HapMap mitochondrial SNPs. Two pedigrees could have likely explained this relationship. Panel A demonstrates a half-sibling relationship, where they have the same mother but different fathers. Panel B shows the two in an avuncular relationship. The position of the NA19192 pedigree could have been swapped with that of NA19130 in this case. Shaded symbols indicated individuals with shared mitochondrial inheritance. (0.34 MB DOC) [file pone.0006711.s004.tif]

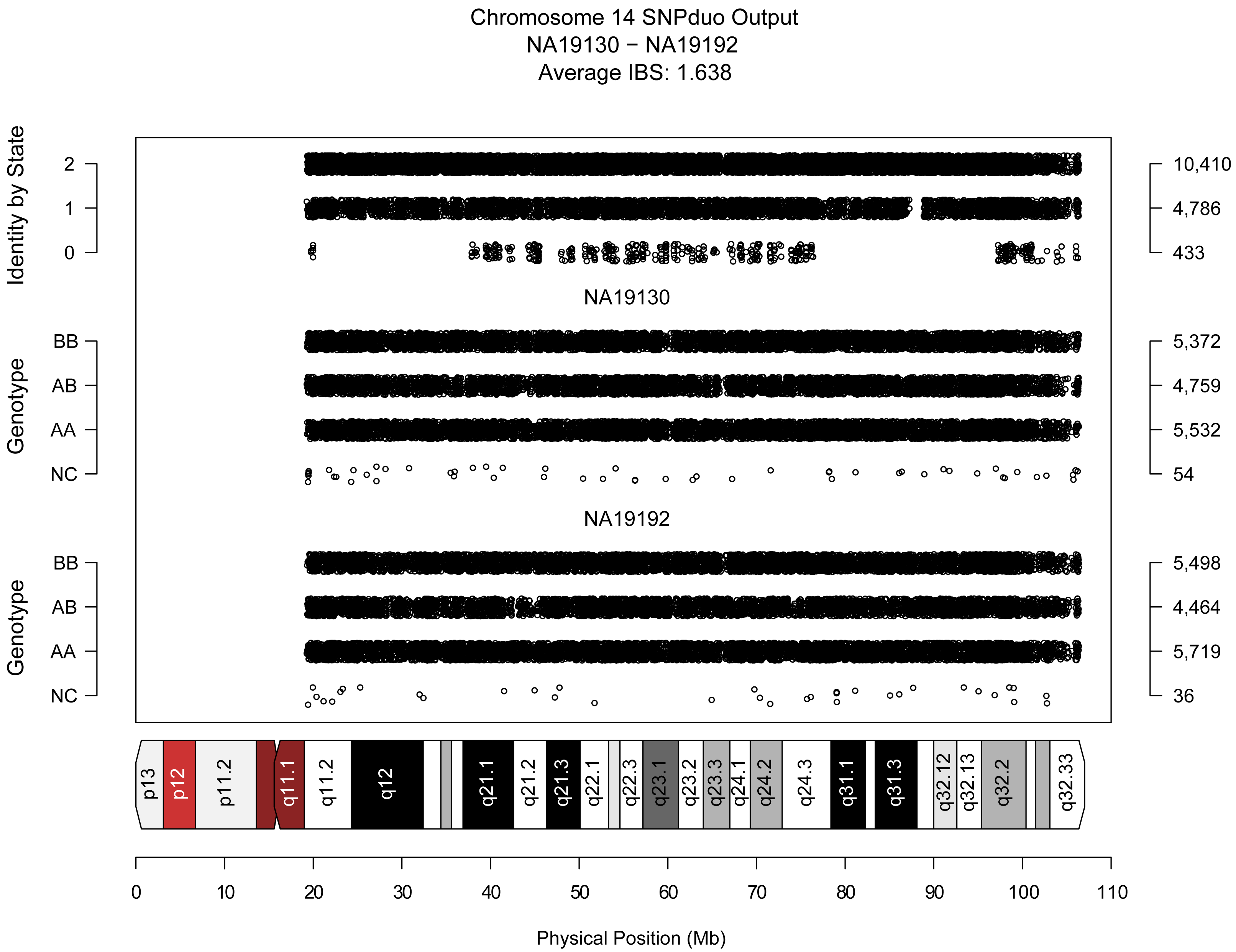

Supplement: Figure S4 — SNPduo output demonstrating variable allele sharing between HapMap individuals NA19130 and NA19192 (genotyped on the Affymetrix 500K platform). Segmental sharing of one allele is observed for chromosome 14. An area of IBS-2 can be seen near the distal end of the q-arm. (0.82 MB DOC) [file pone.0006711.s005.tif]

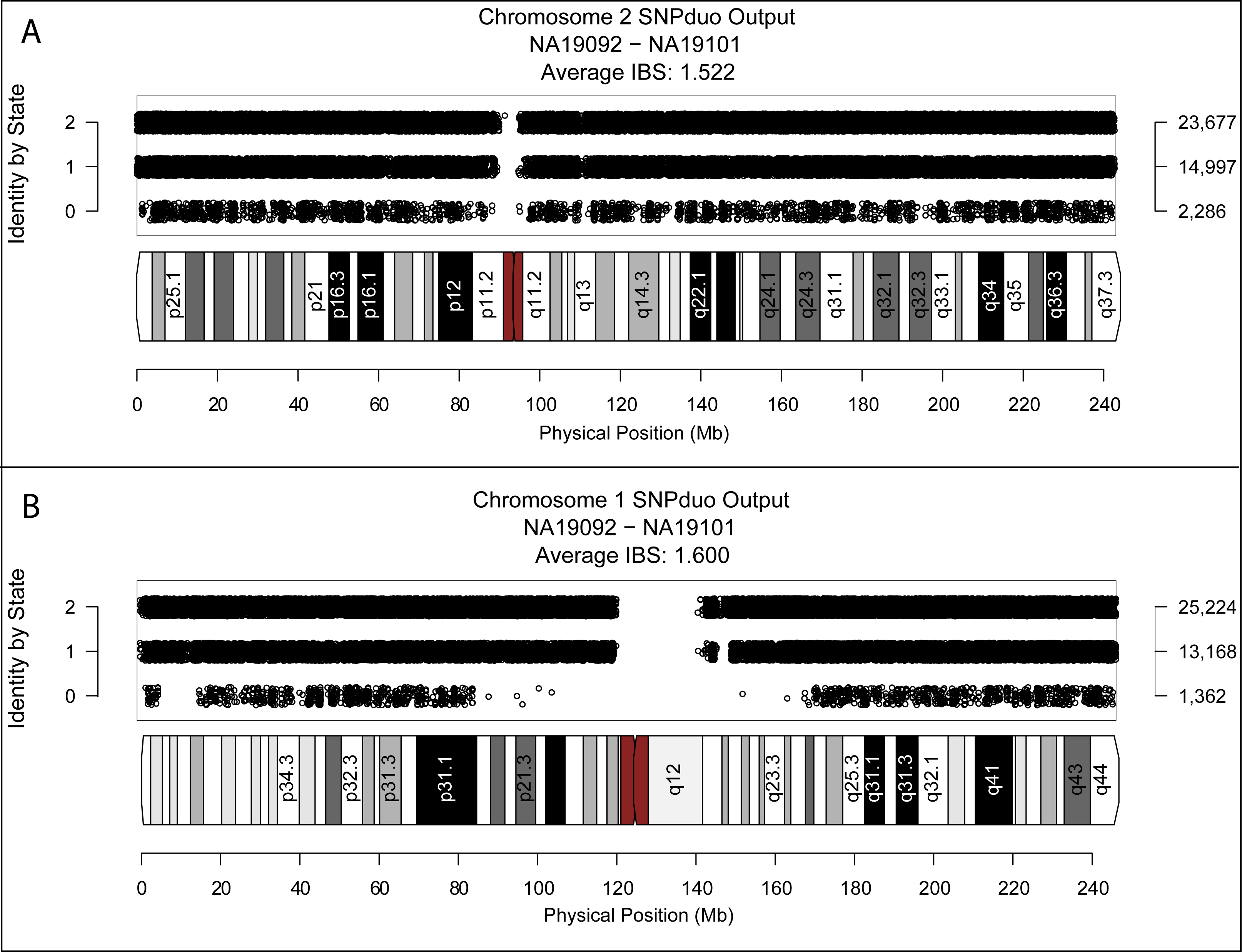

Supplement: Figure S5 — Variable allele sharing from no shared alleles (panel A) to segmental sharing of one allele (panel B). SNPduo output is shown for YRI HapMap individuals NA19092 and NA19101 genotyped on the Affymetrix 500K platform. (A) Chromosome 2 is an example of the two individuals sharing no inherited DNA for the entire length of a chromosome. (B) Chromosome 1 demonstrated variable sharing of one allele across an entire chromosome as well as regions of no shared alleles. (0.93 MB DOC) [file pone.0006711.s006.tif]

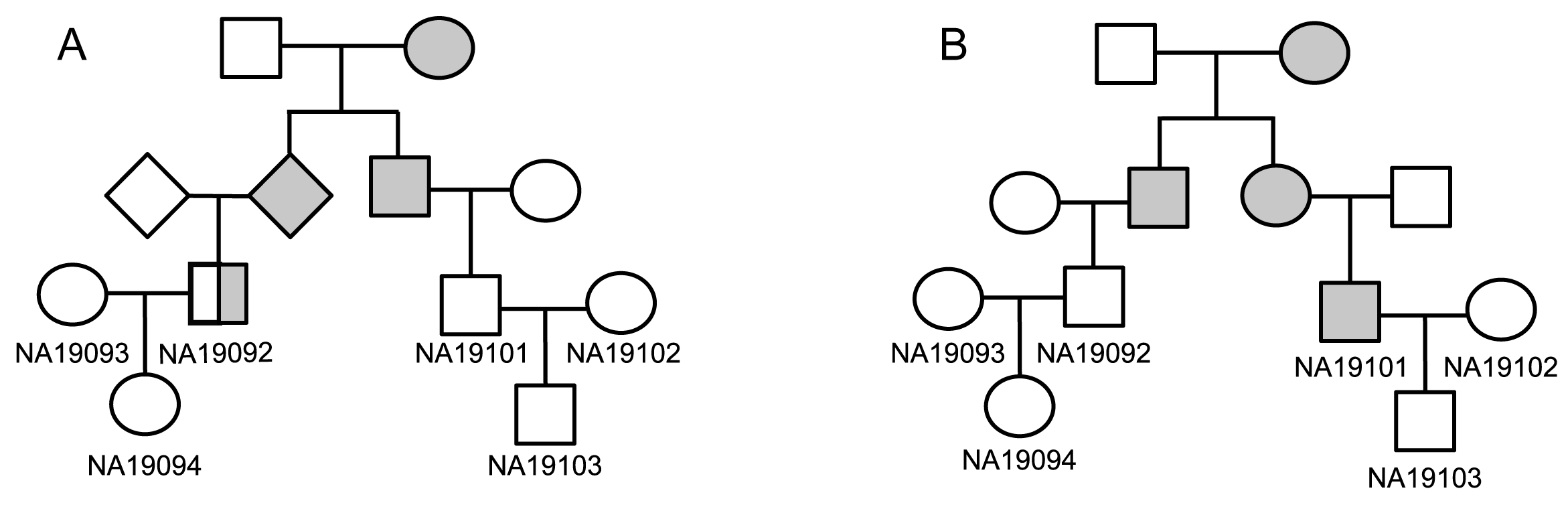

Supplement: Figure S6 — Inferred pedigrees for HapMap NA19092 and NA19101. Shown are two possible pedigree configurations that would explain the relationship between NA19093 and NA19101 if they are first cousins. Panels A and B are two configurations for a first cousin relationship. Both configurations took into account the lack of a shared mitochondrial haplotype. In this figure diamonds indicate individuals where the sex of the individual is irrelevant in the context of the mitochondrial inheritance pattern. Shaded symbols had common mitochondrial inheritance. A half-shaded symbol may or may not have shared mitochondrial lineage based on ambiguous parental assignment in the previous generation. (0.20 MB DOC) [file pone.0006711.s007.tif]

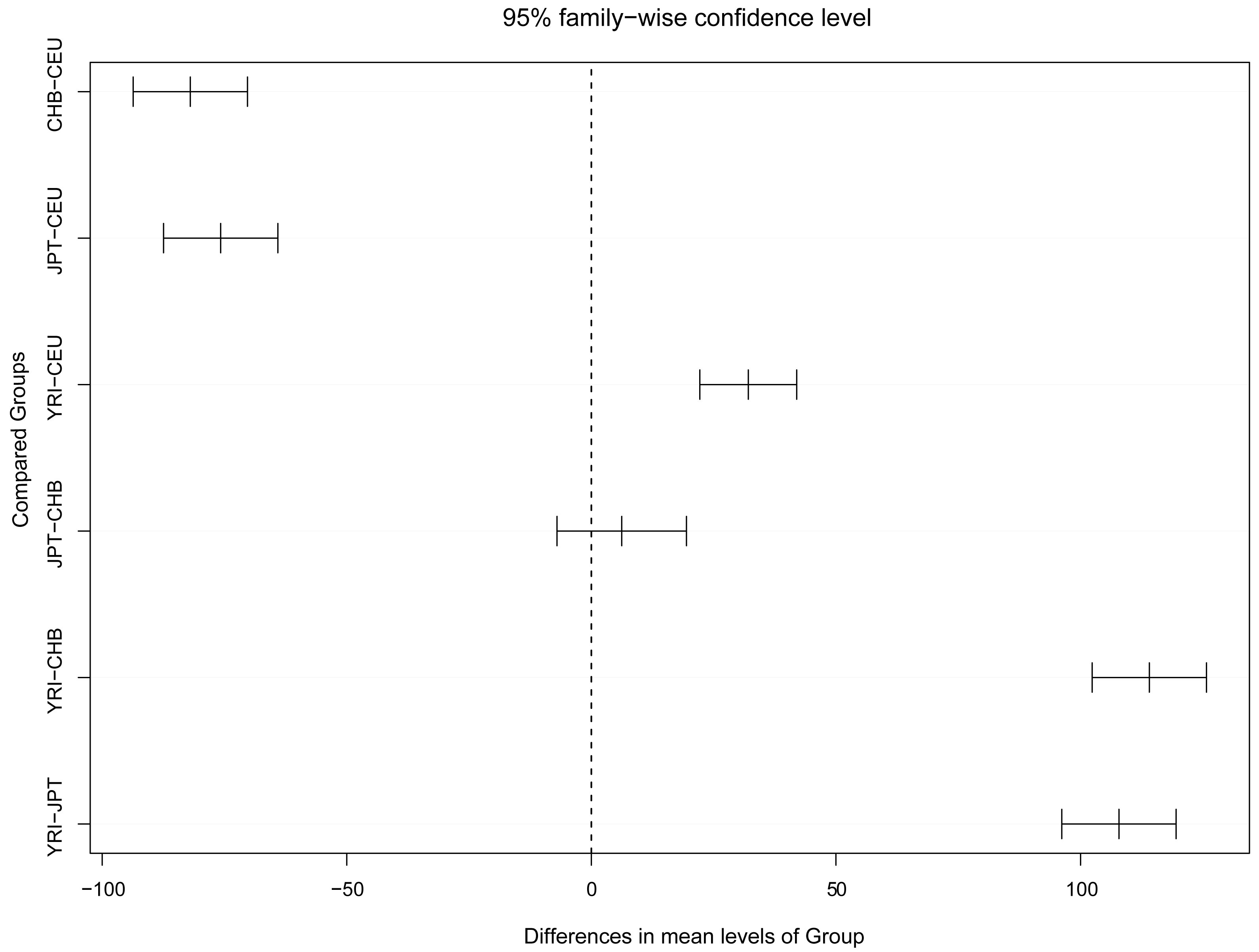

Supplement: Figure S7 — A Tukey Honest Simple Differences boxplot showed differences in the number of autosomal IBS-0 counts for within group comparisons of the four original HapMap groups. The Tukey Honest Simple Differences were plotted for the difference by group in the number of IBS-0 classifications for all within-group comparisons. The YRI group demonstrated more diversity than CEU, CHB, or JPT groups as determined by the number of IBS-0 counts between unrelated individuals in that population. The CHB and JPT groups demonstrated the lowest diversity, but with no difference in the mean diversity between them. (0.51 MB DOC) [file pone.0006711.s008.tif]

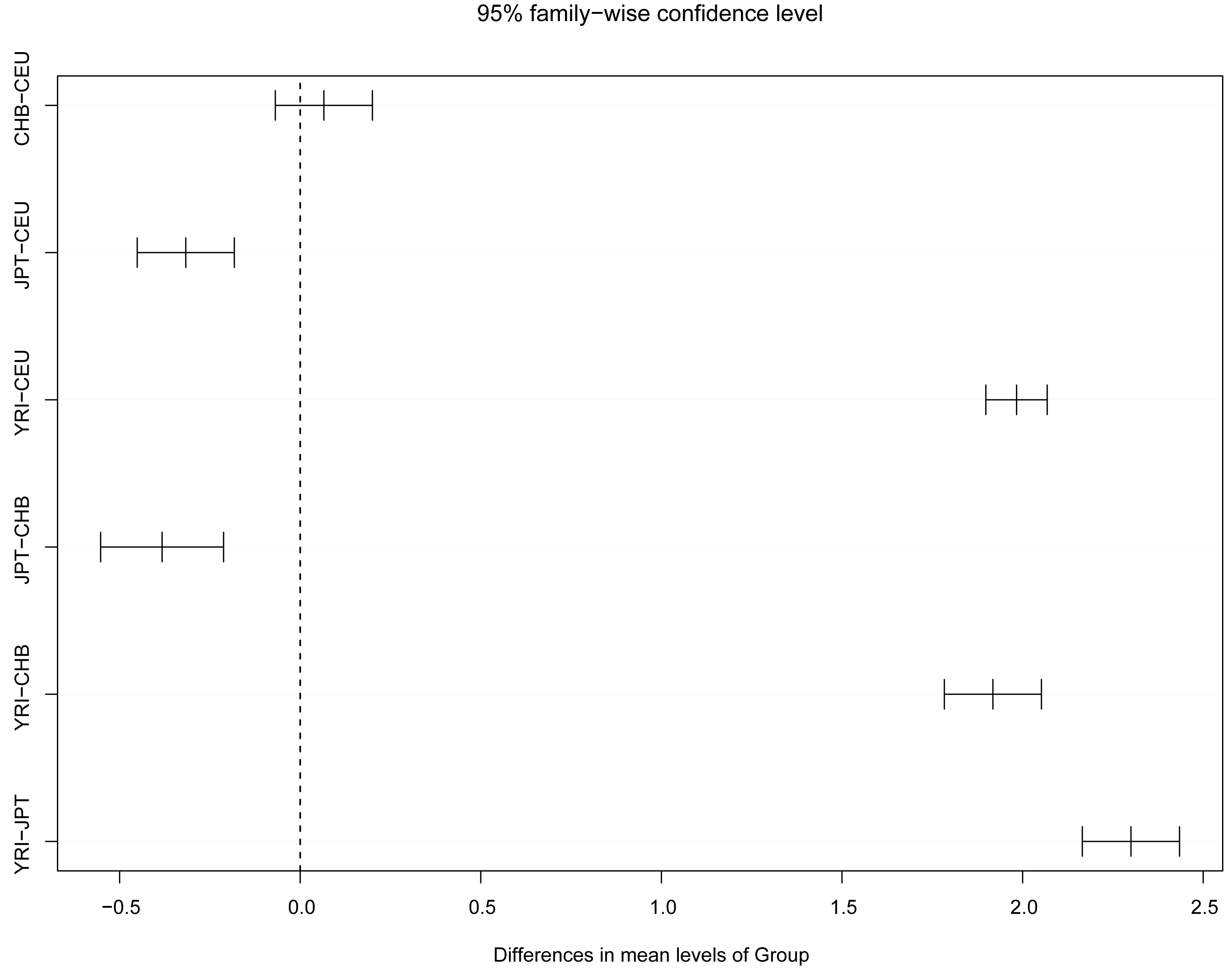

Supplement: Figure S8 — Tukey HSD boxplot of the number of IBS-0 counts for within-group comparisons of mitochondrial SNPs for the four original HapMap groups. The Tukey Honest Simple Differences were calculated for the square-root of the mitochondrial IBS-0 count amongst all unrelated individuals. Mitochondrial genotypes were downloaded from the HapMap project website. The YRI group showed an increased amount of SNP diversity as compared to the CEU, CHB, and JPT groups. The JPT group demonstrated the lowest amount of mitochondrial SNP diversity between unrelated individuals. The CEU and CHB groups showed greater diversity than the JPT group and less than the YRI group, but no difference in the mean number of IBS-0 counts between them. (0.49 MB DOC) [file pone.0006711.s009.tif]

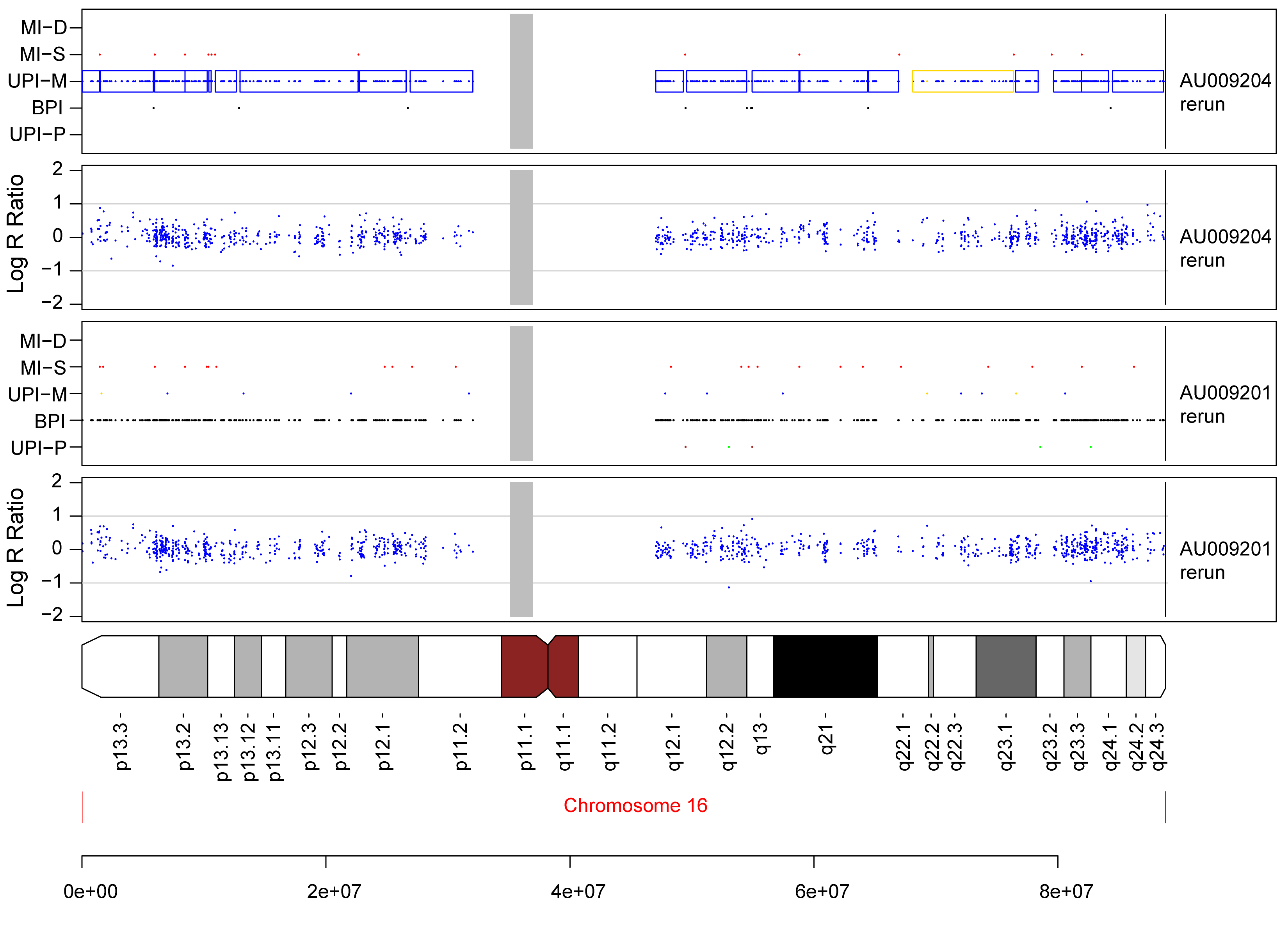

Supplement: Figure S9 — Identification of UPD 16 in the AGRE autism dataset. Shown is SNPtrio output for AU009204 (rerun) and AU009201 (rerun) compared with the detected parents on chromosome 16 (Illumina data). While AU09201 demonstrates routine biparental inheritance for the entire chromosome, AU09204 demonstrates maternal uniparental heterodisomy for the entire chromosome. Both individuals show a nominal Log R Ratio, indicating that the pattern cannot be explained by copy number variation. (0.65 MB DOC) [file pone.0006711.s010.tif]

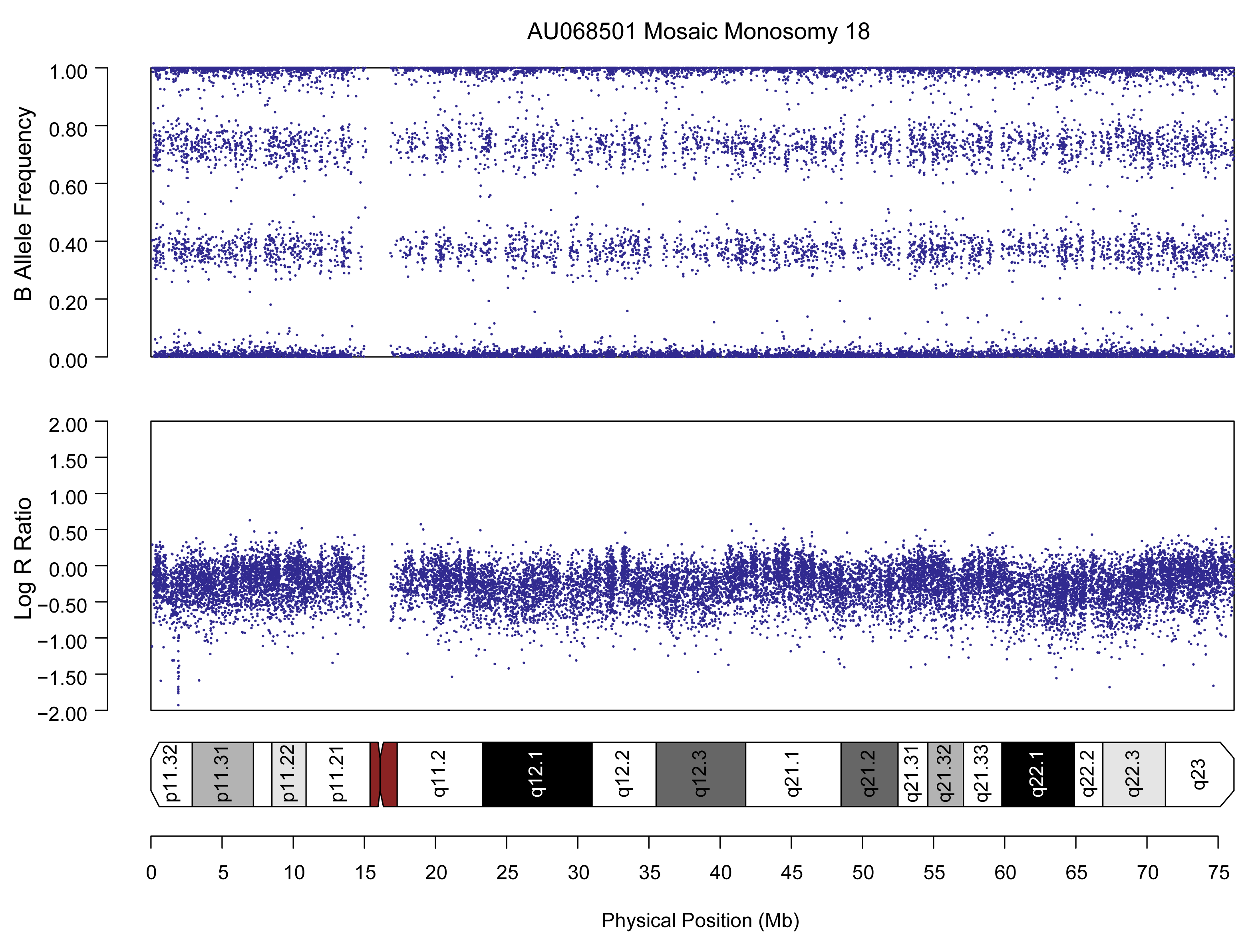

Supplement: Figure S10 — Inferred pedigrees for AGRE mislabeled samples: identification of mosaic monosomy18 in an AGRE proband. Chromosome 18 Illumina marker data are shown for AU068501. The top panel contains the B Allele Frequency data (genotype data), and the bottom panel contains the Log R Ratio (intensity data). The entire chromosome exhibits a dip in Log R Ratio, indicating a loss of DNA. If the sample had 100% monosomy then the corresponding B Allele Frequency would split between 1 and 0. However, the upper panel is split into four tracks, indicating a mosaic monosomy of chromosome 18. (0.98 MB DOC) [file pone.0006711.s011.tif]

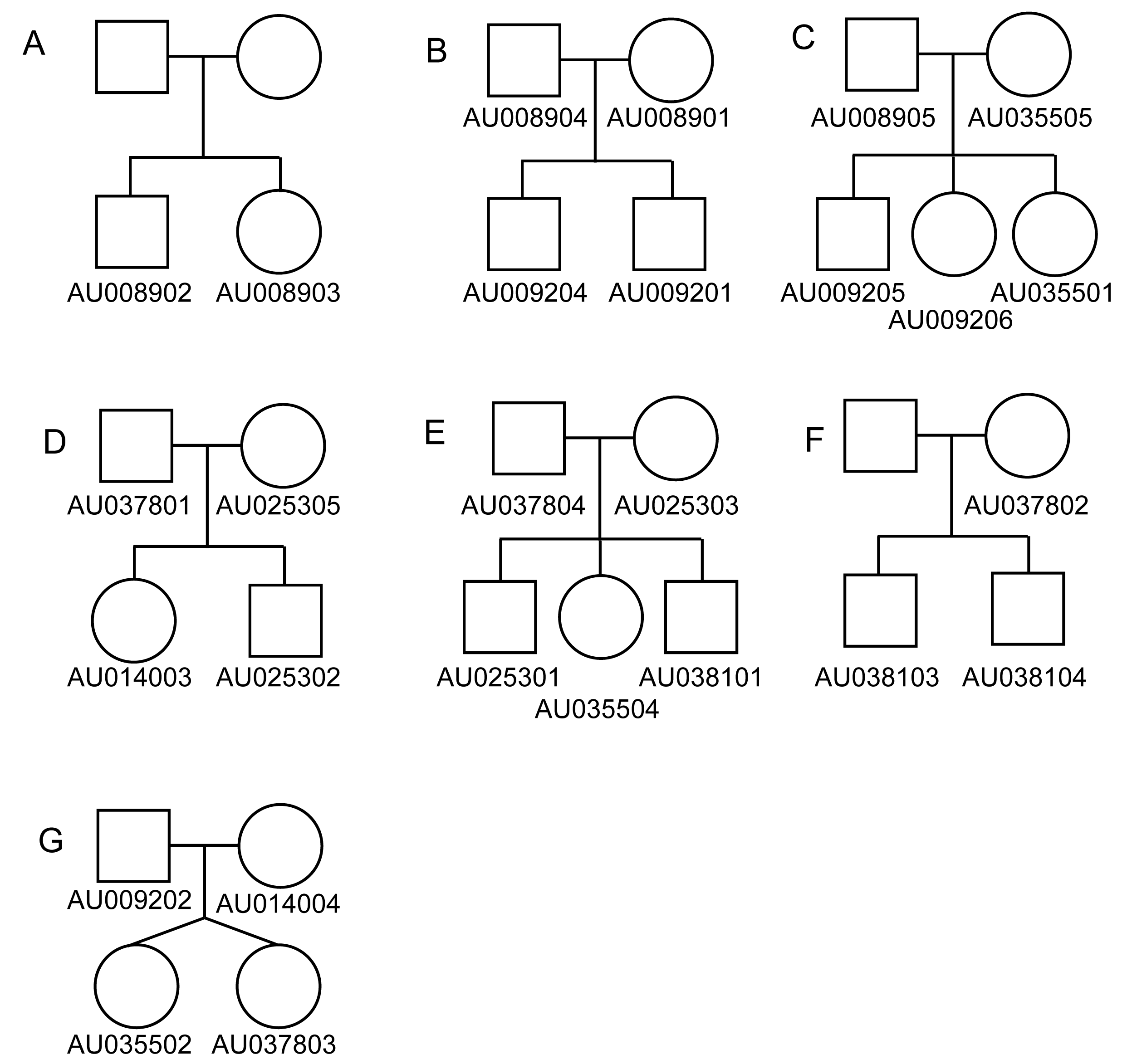

Supplement: Figure S11 — We reconstructed pedigrees from AGRE autism data (Illumina platform). For apparently mislabeled samples we used SNPduo to derive mean and standard deviation of IBS values, then inferred relationships of individuals. The data demonstrate that the rerun samples do not correspond to the original sample ID. However, the true sample ID in each case is unknown. (0.56 MB TIF) [file pone.0006711.s012.tif]
