# Supplementary material for: Visualization of Shared Genomic Regions and Meiotic Recombination in High-Density SNP Data
Source: PLoS One. 2009 Aug 21;4(8):e6711. doi: 10.1371/journal.pone.0006711 (PMC2725774; doi:10.1371/journal.pone.0006711)
Supplement: Table S1 — The samples shown are those identified as identical to each other. These are mostly identical siblings, with the exception of family CEPH, individual 1 and family 12144, individual 12144. These were identical samples labeled as independent individuals. Abbreviations: FID = Family ID. IID = Individual ID. SD = Standard Deviation. Specified = Relationships from ped file. Calculated = Determined from Mean / SD. (0.06 MB DOC) [file pone.0006711.s013.doc]

**Supplementary Table 1. Identical Affymetrix genotyped autism samples**

| **FID1** | **IID1** | **FID2** | **IID2** | **IBS0** | **IBS1** | **IBS2** | **Mean IBS** | **SD IBS** | **Specified** | **Calculated** |
| --- | --- | --- | --- | --- | --- | --- | --- | --- | --- | --- |
| AU0001 | 1JZH | AU0001 | 1JZI | 1 | 2,328 | 394,009 | 1.994120 | 0.076481 | Sibling | Identical |
| AU0001 | 1JZH | AU0001 | 1JZJ | 0 | 220 | 397,299 | 1.999450 | 0.023519 | Sibling | Identical |
| AU0001 | 1JZH | AU0001 | 1JZK | 0 | 43 | 398,461 | 1.999890 | 0.010387 | Sibling | Identical |
| AU0001 | 1JZI | AU0001 | 1JZJ | 1 | 2,325 | 393,270 | 1.994120 | 0.076503 | Sibling | Identical |
| AU0001 | 1JZI | AU0001 | 1JZK | 1 | 2,322 | 394,091 | 1.994140 | 0.076376 | Sibling | Identical |
| AU0001 | 1JZJ | AU0001 | 1JZK | 0 | 216 | 397,371 | 1.999460 | 0.023302 | Sibling | Identical |
| AU0027 | 1K8Y | AU0027 | 1K8Z | 0 | 32 | 398,234 | 1.999920 | 0.008963 | Sibling | Identical |
| AU0030 | 1JWK | AU0030 | 1JWL | 7 | 337 | 394,951 | 1.999110 | 0.030374 | Sibling | Identical |
| AU0075 | 1KBI | AU0075 | 1KBJ | 1 | 56 | 398,118 | 1.999850 | 0.012275 | Sibling | Identical |
| AU0115 | 1KGU | AU0115 | 1KGW | 1 | 5,810 | 385,347 | 1.985140 | 0.121007 | Sibling | Identical |
| AU0132 | 1KRS | AU0132 | 1KH3 | 9 | 852 | 395,860 | 1.997810 | 0.047260 | Sibling | Identical |
| AU0190 | 1KJJ | AU0190 | 1KJK | 0 | 108 | 397,767 | 1.999730 | 0.016473 | Sibling | Identical |
| AU0222 | 1K8G | AU0222 | 1K8H | 2 | 135 | 397,789 | 1.999650 | 0.018954 | Sibling | Identical |
| AU0227 | 1K8U | AU0227 | 1K8V | 0 | 73 | 398,110 | 1.999820 | 0.013539 | Sibling | Identical |
| AU0265 | 1JWG | AU0265 | 1JWF | 0 | 67 | 397,460 | 1.999830 | 0.012981 | Sibling | Identical |
| AU0325 | 1K93 | AU0325 | 1K95 | 0 | 59 | 398,218 | 1.999850 | 0.012170 | Sibling | Identical |
| AU0334 | 1K9M | AU0334 | 1K9N | 0 | 49 | 398,398 | 1.999880 | 0.011089 | Sibling | Identical |
| AU0355 | 1JX8 | AU0355 | 1JX7 | 0 | 109 | 397,222 | 1.999730 | 0.016561 | Sibling | Identical |
| AU0371 | 1K7K | AU0371 | 1K7L | 13 | 1,612 | 393,679 | 1.995860 | 0.064748 | Sibling | Identical |
| AU0386 | 1K7F | AU0386 | 1K7H | 39 | 2,011 | 393,012 | 1.994710 | 0.073873 | Sibling | Identical |
| AU0579 | 1JYH | AU0579 | 1JYI | 0 | 114 | 397,973 | 1.999710 | 0.016920 | Sibling | Identical |
| AU1505 | 1IUG | AU1505 | 1IVF | 1 | 241 | 394,188 | 1.999380 | 0.024915 | Sibling | Identical |
| AU1567 | 1IZR | AU1567 | 1IZS | 8 | 548 | 392,936 | 1.998570 | 0.038366 | Sibling | Identical |
| AU1579 | 1JA7 | AU1579 | 1JA6 | 0 | 34 | 398,839 | 1.999910 | 0.009232 | Sibling | Identical |
| AU1585 | 1J9E | AU1585 | 1J9D | 4 | 151 | 398,546 | 1.999600 | 0.020462 | Sibling | Identical |
| AU1627 | 1JFO | AU1627 | 1JFP | 0 | 328 | 396,657 | 1.999170 | 0.028732 | Sibling | Identical |
| CEPH | 1 | NA12144 | NA12144 | 2 | 137 | 398,468 | 1.999650 | 0.019069 | Unrelated | Identical |
