# Supplementary material for: Visualization of Shared Genomic Regions and Meiotic Recombination in High-Density SNP Data
Source: PLoS One. 2009 Aug 21;4(8):e6711. doi: 10.1371/journal.pone.0006711 (PMC2725774; doi:10.1371/journal.pone.0006711)
Supplement: Table S2 — Listed in this table are individuals the pedigree file specified as being unrelated, but appear to be first degree relatives by IBS analysis. While differentiating between different degrees of relatives can at times be difficult due to genotyping errors, missing calls, and varying population allele frequencies, it is almost always trivial to discriminate between unrelated individuals and first degree relatives in any large dataset. Abbreviations: FID = Family ID. IID = Individual ID. SD = Standard deviation. Specified = Relationship specified in pedigree information. Calculated = Relationship calculated from the Mean and SD of IBS. (0.05 MB DOC) [file pone.0006711.s014.doc]

Supplementary Table 2. Unspecified related individuals

| **FID1** | **IID1** | **FID2** | **IID2** | **IBS0** | **IBS1** | **IBS2** | **Mean IBS** | **SD IBS** | **Specified** | **Calculated** |
| --- | --- | --- | --- | --- | --- | --- | --- | --- | --- | --- |
| AU0052 | 1IJS | AU0052 | 1LAO | 11,429 | 119,145 | 265,006 | 1.64103 | 0.53656 | Unrelated | Sibling |
| AU0052 | 1IJU | AU0052 | 1LAO | 12,451 | 120,956 | 262,273 | 1.63137 | 0.54376 | Unrelated | Sibling |
| AU0052 | 1IJV | AU0052 | 1LAO | 13,362 | 122,227 | 260,116 | 1.62358 | 0.54978 | Unrelated | Sibling |
| AU0052 | 1IJW | AU0052 | 1LAR | 11,958 | 123,475 | 260,784 | 1.62800 | 0.54220 | Unrelated | Sibling |
| AU0081 | 1K64 | AU0081 | 1I3N | 9,885 | 131,766 | 255,627 | 1.61856 | 0.53452 | Unrelated | Sibling |
| AU0081 | 1K66 | AU0081 | 1I3O | 10,820 | 128,182 | 257,977 | 1.62259 | 0.53804 | Unrelated | Sibling |
| AU0180 | 1I7J | AU0180 | 1IFR | 10,831 | 132,705 | 254,981 | 1.61265 | 0.54006 | Unrelated | Sibling |
| AU0186 | 1K86 | AU0049 | 1K88 | 60 | 120,922 | 277,124 | 1.69596 | 0.46033 | Unrelated | ParentChild |
| AU0186 | 1K86 | AU0049 | 1K89 | 49 | 122,068 | 276,085 | 1.69321 | 0.46143 | Unrelated | ParentChild |
| AU0264 | 1JTD | AU0264 | 1JPK | 10,691 | 133,077 | 254,091 | 1.61177 | 0.53968 | Unrelated | Sibling |
| AU0264 | 1JTD | AU0264 | 1JPM | 10,426 | 132,331 | 252,885 | 1.61282 | 0.53849 | Unrelated | Sibling |
| AU0603 | 1IL5 | AU0603 | 1I44 | 10,057 | 130,030 | 257,311 | 1.62218 | 0.53450 | Unrelated | Sibling |
| AU0795 | 1IBS | AU0795 | 1IBU | 106 | 121,838 | 275,204 | 1.69268 | 0.46196 | Unrelated | ParentChild |
| AU1072 | 1K2X | AU1072 | 1IGV | 11,241 | 121,871 | 260,905 | 1.63364 | 0.53777 | Unrelated | Sibling |
| AU1072 | 1K2Y | AU1072 | 1IGY | 11,386 | 122,388 | 262,389 | 1.63359 | 0.53818 | Unrelated | Sibling |
| AU1072 | 1K31 | AU1072 | 1IGY | 11,575 | 126,187 | 258,730 | 1.62335 | 0.54145 | Unrelated | Sibling |
| AU1072 | 1K32 | AU1072 | 1IGY | 11,599 | 122,899 | 262,184 | 1.63170 | 0.53957 | Unrelated | Sibling |
| AU1078 | 1IHC | AU1078 | 1IHF | 12,440 | 129,667 | 253,046 | 1.60889 | 0.54873 | Unrelated | Sibling |
| AU1222 | 1KY9 | AU1222 | 1KXP | 6,287 | 101,296 | 287,418 | 1.71172 | 0.48683 | Unrelated | Sibling |
| AU1469 | 1JJQ | AU1469 | 1JJW | 3,894 | 123,730 | 254,736 | 1.65604 | 0.49601 | Unrelated | Sibling |
