# Supplementary material for: Visualization of Shared Genomic Regions and Meiotic Recombination in High-Density SNP Data
Source: PLoS One. 2009 Aug 21;4(8):e6711. doi: 10.1371/journal.pone.0006711 (PMC2725774; doi:10.1371/journal.pone.0006711)
Supplement: Table S3 — Crossover detection schema. Four individuals are required: a child, a parent, and the grandparents on the chosen parent's side. Only homozygous child SNPs that correspond to heterozygous parental SNPs can be informative. Each of the informative SNP types can conclusively link one allele to a particular grandparent. Abbreviations: GP: Grandpaternal, GM: Grandmaternal, PA: Parent, CH: child, GP-T: Grandpaternal-type, GM-T: Grandmaternal-type. (0.04 MB DOC) [file pone.0006711.s015.doc]

**Supplementary Table 3.**

| **GP** | **GM** | **PA** | **CH** | **GP-T** | **GM-T** |
| --- | --- | --- | --- | --- | --- |
| AA | AB | AB | AA | X |  |
| AA | AB | AB | BB |  | X |
| AA | BB | AB | AA | X |  |
| AA | BB | AB | BB |  | X |
| AB | AA | AB | AA |  | X |
| AB | AA | AB | BB | X |  |
| AB | BB | AB | AA | X |  |
| AB | BB | AB | BB |  | X |
| BB | AA | AB | AA |  | X |
| BB | AA | AB | BB | X |  |
| BB | AB | AB | AA |  | X |
| BB | AB | AB | BB | X |  |
